# Impact of Maternal Obesity on the Gestational Metabolome and Infant Metabolome, Brain, and Behavioral Development in Rhesus Macaques

**DOI:** 10.3390/metabo12080764

**Published:** 2022-08-19

**Authors:** Yu Hasegawa, Zhichao Zhang, Ameer Y. Taha, John P. Capitanio, Melissa D. Bauman, Mari S. Golub, Judy Van de Water, Catherine A. VandeVoort, Cheryl K. Walker, Carolyn M. Slupsky

**Affiliations:** 1 Department of Food Science and Technology, University of California-Davis, Davis, CA 95616, USA; 2 California National Primate Research Center, University of California-Davis, Davis, CA 95616, USA; 3 The UC Davis MIND Institute, University of California-Davis, Sacramento, CA 95817, USA; 4 Department of Psychiatry and Behavioral Sciences, University of California-Davis, Sacramento, CA 95817, USA; 5 Department of Internal Medicine, University of California-Davis, Sacramento, CA 95817, USA; 6 Department of Obstetrics and Gynecology, University of California-Davis, Davis, CA 95616, USA; 7 Department of Nutrition, University of California-Davis, Davis, CA 95616, USA

**Keywords:** obesity, pregnancy, infant development, metabolomics, NMR, urine, plasma, placenta, brain, behavior

## Abstract

Maternal gestational obesity is associated with elevated risks for neurodevelopmental disorder, including autism spectrum disorder. However, the mechanisms by which maternal adiposity influences fetal developmental programming remain to be elucidated. We aimed to understand the impact of maternal obesity on the metabolism of both pregnant mothers and their offspring, as well as on metabolic, brain, and behavioral development of offspring by utilizing metabolomics, protein, and behavioral assays in a non-human primate model. We found that maternal obesity was associated with elevated inflammation and significant alterations in metabolites of energy metabolism and one-carbon metabolism in maternal plasma and urine, as well as in the placenta. Infants that were born to obese mothers were significantly larger at birth compared to those that were born to lean mothers. Additionally, they exhibited significantly reduced novelty preference and significant alterations in their emotional response to stress situations. These changes coincided with differences in the phosphorylation of enzymes in the brain mTOR signaling pathway between infants that were born to obese and lean mothers and correlated with the concentration of maternal plasma betaine during pregnancy. In summary, gestational obesity significantly impacted the infant systemic and brain metabolome and adaptive behaviors.

## 1. Introduction

The prevalence of obesity has been steadily increasing [1], with more than half of women of reproductive age in the United States ebing overweight, and about a third clinically obese [2]. Evidence from the CHARGE (CHildhood Autism Risks from Genetics and the Environment) population-based case-control study revealed that children with autism spectrum disorder (ASD) were more likely to have mothers with obesity [3]. Obesity is associated with an elevated risk of developing Type 2 diabetes mellitus, and infants of diabetic mothers show a greater risk of impairment in recognition memory performance that is regulated by the hippocampus [4]. Despite growing epidemiological evidence, the mechanisms by which maternal obesity may lead to behavioral changes in offspring remain elusive. 

Brain development occurs from the first trimester of gestation and continues after birth, increasing the complexity in physical structure, connectivity, and functional capacity of brain [5]. Although some fundamental behavioral functions are present at birth (e.g., hearing, touch/pain sensation), more complex behaviors, such as recognition memory, are developed after birth as a result of the rapid growth of neural circuits and connectivity, especially in the hippocampus [5]. One of the critical pathways that is involved in the anatomical and functional development of the brain is the mechanistic target of rapamycin (mTOR) signaling pathway. mTOR is a serine/threonine protein kinase that is composed of two protein complexes, mTORC1 and mTORC2 [6]. The mTOR pathway is the master regulator of a large number of major cellular functions including protein synthesis, as well as cellular growth and proliferation in response to both intracellular and extracellular cues [6]. The mTOR pathway plays a role in regulating neuronal and glial differentiation, neural stem cell function [7], homeostasis of synaptic protein synthesis, and degradation by autophagy [8], which are critical for neurodevelopment [9]. This may be of significance in obese populations, as the incidence of high blood glucose that is induced by insulin insensitivity is greater in obese compared to lean individuals [10]. Higher maternal blood glucose leads to higher fetal blood glucose, which stimulates insulin secretion from the fetal pancreas [11]. It is plausible that in this environment, fetal brain mTORC1 activity is increased via the mTOR-insulin signaling pathway [6], which could subsequently lead to aberrant cognitive development. Indeed, mTOR dysregulation has been implicated in various brain disorders including ASD and learning disability [7,12], and therefore, the regulation of mTOR activation to an appropriate level is important for neurogenesis in brain development. 

To gain a better understanding of the mechanistic pathways between maternal obesity and offspring metabolism and cognition, we performed a longitudinal study in obese and lean pregnant nonhuman primates and their infants. We hypothesized that mothers with obesity would show altered systemic and placental metabolism during pregnancy, which would lead to dysregulation in development and be reflected in the serum and brain metabolome of the offspring. A unique aspect of our study was that obesity was not induced by a specific diet, but rather was a consequence of overnutrition and low activity in the individual monkeys, and thus is highly relevant to obese humans. Our results suggest that gestational maternal obesity significantly impacted the infant systemic and brain metabolome, as well as adaptive behaviors.

## 2. Materials and Methods

### 2.1. Study Population

Animal handling was approved by the University of California-Davis Institutional Animal Care and Use Committee (IACUC protocol#19299) and all the experiments were performed in accordance with the relevant guidelines and regulations.

Pregnant female rhesus macaques (*Macaca mulatta*) with appropriate social behavior and a previous successful pregnancy were selected from an indoor breeding colony at the California National Primate Research Center (CNPRC, Davis, CA, USA). Fetal sex was determined by a qualitative real-time PCR assay to detect the Y chromosome [13] and only those with male fetuses were chosen for this study. The animals that were used in this study had maintained a consistent body condition score (BCS) for at least one year prior to the study. Obesity is defined when subjects have body fat above 30% for women (American Medical Association, Chicago, IL, USA). A BCS of 3.5 was chosen as the cut off for inclusion in the obese group for this study as a BCS of 3.5 is correlated with 32% body fat [14]. Mothers with a BCS of 2.5 or lower comprised the Lean group. The sample size of the biological samples was not balanced due to fetal deaths for unknown reasons, misidentification of female offspring, technical issues in collecting enough sample volume for analysis, or the recruitment of additional animals into the study in the middle of pregnancy to account for the sample loss (Supplementary Table S1). The final number of mothers and their offspring included six for the Lean and seven for the Obese groups (Supplementary Table S2). 

### 2.2. Feeding and Rearing of Animals

Adult animals were fed seven biscuits (#5047; LabDiet, St. Louis, MO, USA) twice daily between 6–9 a.m. and 1–3 p.m. All the mothers were provided nine biscuits twice daily once pregnancy was determined, and twelve biscuits twice daily while nursing infants that were 4 months and older. Fresh produce was provided bi-weekly and water was available ad libitum. A more detailed description is available in Appendix A.

### 2.3. Sample Collection and Processing

All the animals were coded by IDs, and therefore, the animal caretakers and researchers who collected samples and conducted behavioral testing were blinded for the group assignment. The collected biological samples were randomized using random numbers that were generated in R in conducting assessments, and the group assignment was blinded until the data were analyzed. On the day prior to sample collection, food was removed approximately 30 min after the feeding in the afternoon, and biological samples were collected before the morning feeding. Pregnancy in rhesus macaques lasts for 166.5 days on average [15]. Plasma and urine samples were collected from the mothers once during the 1st and 2nd trimesters, and twice during the 3rd trimester on gestational day (GD) 45, 90, 120, and 150 after anesthetizing the animals with 5–30 mg/kg ketamine or 5–8 mg/kg telazol. Blood samples were collected in 5 mL lavender top (EDTA) or green (heparin) tubes and the supernatant was collected. Urine was collected from the bladder by ultrasound-guided transabdominal cystotomy using a 22-gauge needle and subsequently centrifuged to collect the supernatant. Within 15 days prior to delivery, a placental sample was collected transabdominally under ultrasound guidance using an 18-gauge needle that was attached to a sterile syringe and centrifuged to collect the pellet.

Infant plasma was collected at postnatal day (PD) 30, 90, and 110, and plasma, urine, and brain tissues were collected at PD180 when necropsy was conducted between 9:30 a.m. and 1:30 p.m. The infants were anesthetized with ketamine and plasma and urine were collected, followed by euthanasia with 120 mg/kg pentobarbital. Heparin injection and clamping of the descending aorta was followed by flushing with saline at room temperature for 2 min and then by saline at 4 °C for 5 min at 250 mL/min until clear. The brain was extracted, and four regions (amygdala, hippocampus, hypothalamus, and prefrontal cortex) were dissected and immediately frozen. All the collected samples were stored at −80 °C.

### 2.4. Metabolite Extraction and Insulin, Cytokine, and Cortisol Measurement

The plasma and urine samples were thawed on ice and filtered by Amicon Ultra Centrifugal Filter (3k molecular weight cutoff; Millipore, Billerica, MA, USA), and the filtrate was used for metabolomics analysis. The samples were stored at 4 °C overnight and the pH was adjusted to 6.8 ± 0.1. For the placental samples, polar metabolites were extracted as described [16] with the following modification: after lyophilization of the polar metabolite layer, the dried sample was reconstituted in 270 µL of 10 mM phosphate buffer (pH 6.85) that was prepared in deuterium oxide. The samples were transferred to 3 mm Bruker nuclear magnetic resonance (NMR) tubes and kept at 4 °C until NMR data acquisition within 24 h of sample preparation.

A multiplex Bead-Based Kit (Millipore) was used to measure insulin as well as 17 cytokine and chemokine levels in the heparin-treated plasma samples including high-sensitivity C-reactive protein (hs-CRP), Granulocyte-macrophage colony-stimulating factor (GMCSF), interferon-γ (IFN-γ), tumor necrosis factor-α (TNF-α), transforming growth factor-α (TGF-α), monocyte chemoattractant protein-1 (MCP-1), macrophage inflammatory protein-1β (MIP-1β), interleukin (IL)-1β, IL-1 receptor antagonist (IL-1ra), IL-2, IL-6, IL-8, IL-10, IL-12/23 p40, IL-13, IL-15, and IL-17A. The assay was performed following the manufacturer’s protocol. The assessment of infant plasma cortisol was conducted as previously described [17,18]. Briefly, the infants were separated from their mothers at 9 a.m. and blood samples were collected at 11 a.m. (Sample 1). Another blood collection was done at 4 p.m. (Sample 2), followed by intramuscular injection of 500 μg/kg dexamethasone (Dex). Blood was collected at 8:30 a.m. of the following day (Sample 3) and 2.5 IU of adrenocorticotropic hormone (ACTH) was then injected intramuscularly. After 30 min, the last blood was collected (Sample 4). A more detailed description is available in Appendix A.

### 2.5. ^1^H NMR Spectroscopy Data Acquisition

We conducted an untargeted metabolomics analysis using ^1^H NMR spectroscopy. All spectra were acquired at 25 °C using the noesypr1d pulse sequence on a Bruker Avance 600 MHz NMR spectrometer (Bruker, Billerica, MA, USA) [19]. The identification and quantification of metabolites were completed using Chenomx NMRSuite (version 8.1, Chenomx Inc., Edmonton, AB, Canada).

### 2.6. Protein Analysis

Protein was extracted from the cell layer that was collected after metabolite extraction of brain samples, and protein quantification and Western blots were done as previously described [16]. The following antibodies from Cell Signaling Technology (Danvers, MA, USA) were used for the western blots: rabbit anti-Akt (#9272), anti-phospho-Akt (#9275; Thr308), anti-AMPKα (#2603), anti-phospho-AMPKα (#2535; Thr172), anti-p70 S6K (#9202), anti-phospho-p70 S6K (#9234; Thr389), as well as goat anti-rabbit IgG antibody conjugated to horseradish peroxidase (#7074). Either Clarity Western ECL Blotting Substrates (Bio-Rad) or Radiance Plus (Azure biosystems, Dublin, CA, USA) were used depending on the strength of signal for chemiluminescent detection. Refer to Appendix A for more detailed description. 

### 2.7. Visual Paired-Comparison (VPC) Test

Recognition memory was tested with infants on post-conception day 200 ± 3 days using a VPC test that was conducted between 8:30 and 10:30 a.m. [20,21,22]. Briefly, the infants were hand-held in front of a testing booth to look at two identical black and white high contrast abstract pictures (Fagan Test of Infant Intelligence; Infantest Corporation, Cleveland, OH, USA) that were placed to the right and left of center for a total of 20 s of cumulative looking time (familiarization trial). Then, the familiar and novel pictures were placed either right or left of center according to a pre-decided random order for a 10 s test period from the time of the first fixation (preference trial 1). The side of the pictures was switched and a second 10 s test period was conducted (preference trial 2). A total of four problems were presented to each infant. The trials were video recorded for later scoring of frequency and duration of the looking patterns using The Observer software (Noldus, Inc., Wageningen, The Netherlands). Novelty preference was calculated as: the number of fixations at the novel stimulus/the number of fixations at both the novel and familiar stimulus.

### 2.8. Human Intruder (HI) Test

The HI test was conducted as described previously [17]. In short, we examined the frequency of scratch (as an indicator of anxiety [23]) in response to the following four graded levels of stress (1 min each): Profile-Far (technician presented the left profile from ~1 m away from an infant in a cage), Profile-Near (presented left profile from ~0.3 m), Stare-Far (made direct eye contact with the animal from far), and Stare-Near (direct eye contact from near position). 

### 2.9. Statistics

The overall gestational weight gain (GWG) rate was obtained using the following equation: 1000 × ln(W2/W1)/(D2-D1), where D1 is the date the last weight (W1) was obtained before pregnancy and D2 is the date the last weight (W2) was obtained before birth. Homeostatic model assessment for insulin resistance (HOMA-IR) was calculated using fasting glucose (mg/dL) × fasting insulin (μU/mL)/405. The estimated placental volume (EPV) was calculated as follows: (πT/6) × [4H(W−T) + W(W−4T) + 4T^2^], where T is thickness at maximal height, H is height at maximal width, and W is maximal width measured by ultrasound [24].

The metabolite concentrations were log-transformed prior to the application of the following statistics. A linear mixed-effects model was fitted followed by analysis of variance (ANOVA) to test the group difference of samples with multiple time points using the lme4 package (version 1.1.21). Estimated marginal means were obtained using emmeans (version 1.4.4), and a pairwise comparison was applied as a post-hoc test. R^2^ values were obtained as the effect size using r2glmm (version 0.1.2) (medium effect when 0.15 < R^2^ < 0.35; large when 0.35 ≤ R^2^). *t*-tests were used to test for group differences of samples that were collected at a single time point. The effect size was calculated using Cohen’s D (d) measurement (medium effect when 0.5 < d < 0.8; large when 0.8 ≤ d < 1.3; and very large when 1.3 ≤ d). For metabolomics analysis, Benjamini–Hochberg false discovery rate correction was applied to the *p*-values. For post hoc tests, a *p*-value of 0.05 was used as the cut-off. The correlation between HOMA-IR and EPV was assessed by repeated measures correlation using rmcorr package (version 0.4.1) and other correlations were tested by the Pearson or Spearman methods and visualized using ggscatter (version 0.2.5). The correlation coefficient (R) was used as the effect size (medium effect when 0.3 < R < 0.5; large when 0.5 ≤ R ≤ 1). In order to address the small sample size and the inherent large heterogeneity and variability of rhesus macaque data, we utilized a combination of *p*-values < 0.1 and medium-large effect size to define statistical significance [25]. A more detailed description is available in Appendix A.

## 3. Results

### 3.1. Obese Mothers Exhibited Increased Insulin Resistance and Higher Inflammation over the Course of Pregnancy

For this study, a combination of *p*-value < 0.1 and medium to large effect size was defined as statistically significant to account for the small sample size and the inherent variability that was present in rhesus macaque data [25]. The selection of animals for each group was based on the level of adiposity that was estimated from a BCS score ranging from 1 to 5 [14]. Since obesity is defined when women have body fat that is above 30% according to American Medical Association, the Obese group consisted of animals with a BCS of 3.8 ± 0.3 (average body fat = 32.8% [14]), 10.2 ± 1.4 years old, and 10.8 ± 1.2 kg pre-pregnancy weight, whereas the Lean group consisted of animals with a BCS of 2.2 ± 0.4 (average body fat = 19.7% [14]), that were 8.9 ± 1.3 years old, and 6.9 ± 1.1 kg pre-pregnancy weight.

**Figure 1:**
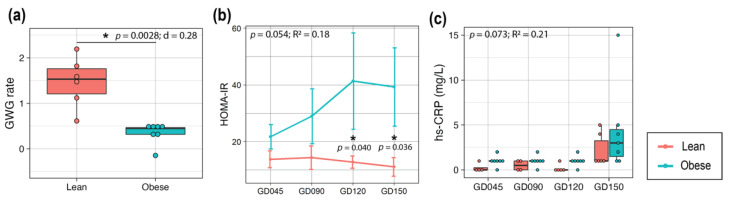
Metabolic characteristics of the Lean and Obese mothers throughout pregnancy. (**a**) GWG rate between the last day before conception and delivery. *t*-tests were used to test for group differences in the overall GWG rate. (**b**) Line plot representing the mean ± SE of HOMA-IR values throughout pregnancy. (**c**) Plasma hs-CRP level. For (**b**,**c**), a linear mixed-effects model was fit followed by ANOVA to test for group differences in HOMA-IR and hs-CRP, and pairwise comparisons were done as post hoc tests using the estimated marginal means. For box plots (**a**,**c**), each dot represents data from an individual animal. Top and bottom of the boxes represent the 25th and 75th percentiles respectively; the middle line represents the median; and the top and bottom whiskers represent maximum and minimum values. Points outside of the whiskers represent outliers. Statistical significance is indicated with “*”. The red and blue lines correspond to the Lean and Obese groups, respectively. Sample size: Lean = 6, Obese = 7. Abbreviations: d or R^2^, effect size measurements; GWG, gestational weight gain; GD, gestational day; HOMA-IR, homeostatic model assessment for insulin resistance; SE, standard error, hs-CRP, high-sensitivity C-reactive protein.

The GWG rate was calculated to assess the steepness of gestational weight change and adjusted by the length of pregnancy when the weights were obtained. The average length of pregnancy was 166 ± 1.4 days (mean ± SEM; Supplementary Table S1), which was typical for rhesus macaques as the average length of pregnancy is 166.5 [15]. Mothers in the Lean group showed a significantly higher GWG rate compared to mothers in the Obese group with a large effect size (*p* = 0.0028, Cohen’s D (d) = 2.8; Figure 1b), suggesting that the Lean group had steeper gestational weight change than the Obese group. Despite the lower GWG, the Obese group became progressively and significantly more insulin resistant compared to the Lean group over the course of pregnancy (*p* = 0.054, R^2^ = 0.18; Figure 1a). In humans, higher levels of insulin resistance have been observed in women with obesity [10,26]. In our study, the Obese group showed a higher coefficient of variation in HOMA-IR compared to the Lean group between GD90-150 (Supplementary Table S3).

Additionally, although 16 plasma cytokines and chemokines that were tested in this study did not show significant differences between the groups (Supplementary Figure S1), the hs-CRP level had a significant group difference showing a higher trend in the Obese group compared to the Lean (*p* = 0.073, R^2^ = 0.21; Figure 1c), which has similarly been observed in human studies [26]. However, the post hoc test did not find a significant group difference.

### 3.2. Maternal Obesity Impacted Energy and One-Carbon Metabolism as Reflected in the Serum and Urine Metabolomes

**Figure 2:**
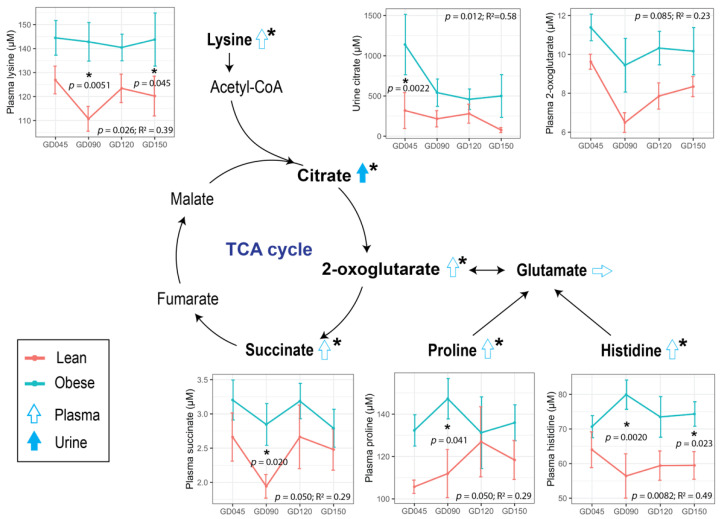
Impact of maternal obesity on energy metabolism that is reflected in the plasma and urine metabolomes during pregnancy. The trends that were found in the Obese group compared to the Lean group are denoted with open blue arrows. Line plots represent the concentrations of plasma or urine metabolites at each time point (mean ± SE). Metabolites that were profiled in this project are in bold. A linear mixed-effects model was fitted followed by ANOVA to test for group difference, and pairwise comparison was done as a post hoc test using the estimated marginal means. The red and blue lines correspond to the Lean and Obese groups, respectively. Statistical significance is indicated with “*”. The sample sizes of the plasma samples are Lean (*n* = 4 at GD45 & GD90, *n* = 5 at GD120, *n* = 6 at GD150) and Obese (*n* = 7); and urine samples are Lean (*n* = 4 at GD45 & GD90 & GD150, *n* = 5 at GD120) and Obese (*n* = 7). Abbreviations: R^2^, effect size measurement; TCA, tricarboxylic acid; GD, gestational day.

**Figure 3:**
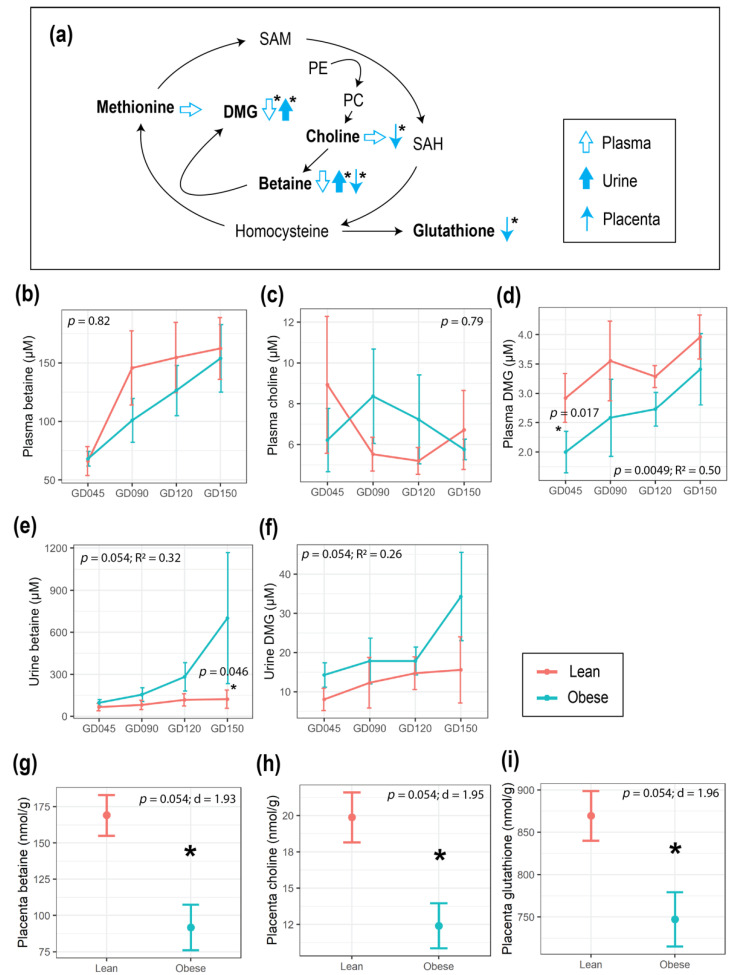
Impact of maternal obesity on one-carbon metabolism that was reflected in the plasma and urine metabolomes during pregnancy. (**a**) Schematic summary of the trends that were found in the metabolites of the one-carbon metabolism pathway, where the arrow indicates an increase (up), decrease (down) or no difference (horizontal) in mothers in the Obese group compared to the Lean group. Plasma is indicated with an open arrow, urine with a solid arrow, and placenta with a thin arrow. Metabolites that were profiled in this study are in bold. (**b**–**i**) Line plots showing the concentration of plasma (**b**) choline, (**c**) betaine, (**d**) DMG; urinary (**e**) betaine, (**f**) DMG; placental (**g**) choline, (**h**) betaine, and (**i**) glutathione (mean ± SE). A linear mixed-effects model was fitted followed by an ANOVA to test the group difference in the concentrations of plasma and urine metabolites, and pairwise comparison was done as a post hoc test using the estimated marginal means. *t*-tests were used to test for group differences in the concentrations of placental metabolites. The red and blue lines correspond to the Lean and Obese groups, respectively. Statistical significance is indicated with “*”. The sample size of plasma samples are: Lean (*n* = 4 at GD45 & GD90, *n* = 5 at GD120, *n* = 6 at GD150) and Obese (*n* = 7); urine samples are Lean (*n* = 4 at GD45 & GD90 & GD150, *n* = 5 at GD120) and Obese (*n* = 7); and placental samples are Lean (*n* = 2) and Obese (*n* = 4). Abbreviations: d or R^2^, effect size measurements; GD, gestational day; DMG, N,N-dimethylglycine; SAM, S-adenosylmethionine; PE, phosphatidylethanolamine; PC, Phosphatidylcholine; SAH, S-adenosylhomocysteine; Eff, effect size; SE, standard error.

To assess the impact of pre-pregnancy obesity on the plasma and urine metabolomes during pregnancy, ^1^H NMR was conducted, and 45 plasma metabolites and 71 urine metabolites were identified and quantified (Supplementary Tables S4 and S5). For mothers in the Obese group, metabolites that are involved in energy metabolism, such as those that are involved in the tricarboxylic acid (TCA) cycle (urinary citrate as well as plasma succinate and 2-oxoglutarate), and amino acids that fuel the TCA cycle (plasma lysine, histidine, and proline), were significantly higher compared to the Lean group throughout gestation (Figure 2). Mothers in the Obese group also showed lower levels of betaine and N,N-dimethylglycine (DMG) in plasma, metabolites that are involved with 1-carbon metabolism, with opposite trends in urine, with statistical significance found in the plasma DMG and urinary betaine and DMG (Figure 3a–f & Supplementary Tables S4 and S5). Our results are concordant with previously published human and animal studies. Glycolysis intermediates pyruvate and citrate in blood were reported to be lower in women with gestational diabetes mellitus during early pregnancy [27] and in tissues of non-pregnant obese mice (both high-fat diet (HFD)-and genetically-induced obesity models) [28]. Also, betaine is a methyl-donor, and lower levels of methyl-donors have been reported in pregnant mothers with obesity and their fetuses [29].

### 3.3. Maternal Obesity Altered One-Carbon Metabolism in the Placenta, and Placental Size Was Correlated with HOMA-IR

**Figure 4:**
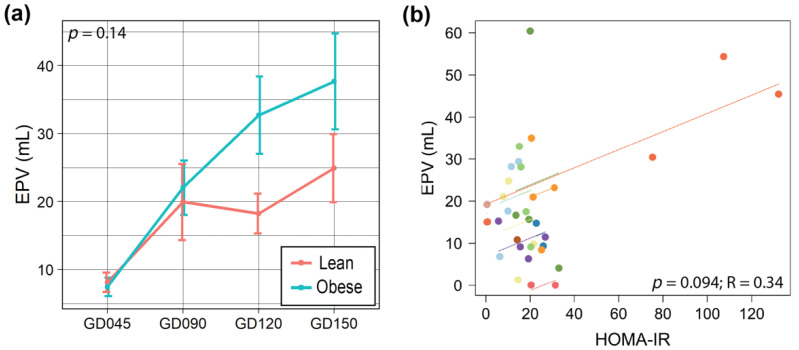
Comparison of the placental size and HOMA-IR in obese and lean mothers. (**a**) Line plot representing EPV over pregnancy. A linear mixed-effects model was fit followed by ANOVA to test for group differences, and a pairwise comparison was done as a post hoc test using the estimated marginal means. The data are expressed as the mean ± SE. (**b**) Repeated measures correlation between EPV and HOMA-IR. Each dot represents data from one time point from an animal. Each line represents a Pearson correlation fit for each animal, and different samples are represented by different colors. The sample size is the same as described in the caption of Figure 3. Abbreviations: R, effect size measurement; GD, gestational day; EPV, estimated placental volume; HOMA-IR, homeostatic model assessment for insulin resistance.

The sample size was small for the placental samples primarily due to technical issues in collecting enough sample volume for analysis (Obese group (*n* = 4), Lean group (*n* = 2)). Due to the rarity of work that has been done on these primate tissues, we included these data as exploratory and urge caution in interpretation. A total of 33 placental metabolites were profiled (Supplementary Table S6). Betaine (*p* = 0.054, d = 1.93), choline (*p* = 0.054, d = 1.95), and their downstream metabolite, glutathione (*p* = 0.054, d = 1.96), were significantly lower in concentration in the placentas from the Obese group with large effect sizes (Figure 3a,g–i). The EPV trended higher in the Obese group compared to the Lean group, although without statistical significance (Figure 4a). Also, EPV showed a significant and moderately positive correlation with HOMA-IR (*p* = 0.093, correlation coefficient (R) = 0.34), which was primarily driven by one Obese mother who had a HOMA-IR level that was above 40 (Figure 4b).

### 3.4. Maternal Obesity Led to Larger Infants at Birth with Altered Metabolomic Profiles

**Figure 5:**
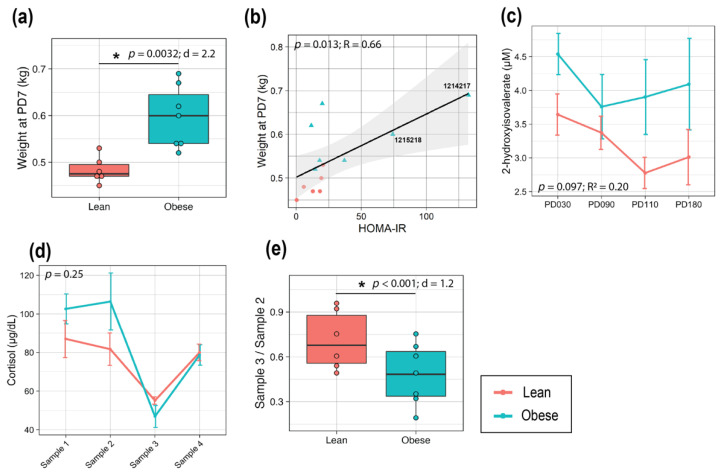
Maternal obesity led to larger infants at birth with altered metabolomic profiles. (**a**) Infant weight at PD7. (**b**) Correlation between maternal HOMA-IR at GD150 and infant weight at PD7. The black line represents the Pearson correlation regression line, and the gray area represents the 95% confidence interval. The seven-digit numbers correspond to Mother ID. Line plots of the concentrations of infant plasma (**c**) 2-hydroxyisovalerate from PD30 to PD180, and (**d**) cortisol over four sampling time points. (**e**) Box plot representing the suppression in plasma cortisol level made by Dex (Sample 3/Sample 2). *t*-tests were used to test for group differences of samples that were collected at a single time point (**a**,**e**) and statistical significance is indicated with a “*”. Data are expressed as the mean ± SE. A linear mixed-effects model was fit followed by ANOVA to test the group difference of samples with multiple time points (**c**,**d**), and pairwise comparisons were done as post hoc tests using the estimated marginal means. For box plots (**a**,**e**), each dot represents data from an individual animal. Top and bottom of the boxes represent the 25th and 75th percentiles respectively; the middle line represents the median; and the top and bottom whiskers represent maximum and minimum values. Points outside of the whiskers represent outliers. Red and blue dots/lines correspond to the Lean and Obese groups, respectively. Sample size is Lean = 6, Obese = 7. Abbreviations: d, R, or R^2^, effect size measurements; PD, postnatal day.

The infant weight that was obtained at PD7 was significantly higher in offspring from mothers in the Obese group compared to the Lean group with a large effect size (*p* = 0.0032, d = 2.2; Figure 5a), as has been observed in human studies [30], and was significantly and positively correlated with maternal HOMA-IR at GD150 (*p* = 0.013, R = 0.66 by Pearson correlation test) (Figure 5b). A larger dispersion of data points that were found in offspring from mothers in the Obese group compared to the Lean group was also observed. Of the four infants with the highest weight at PD7 (normal male infant birthweight is 0.4–0.55 kg [31,32]), two of them (1215218 & 1214217) were from Obese mothers that exhibited very high HOMA-IR values with corresponding metabolic dysregulation: hyperglycemia (fasting glucose > 100 mg/dL [33]), hyperinsulinemia (fasting insulin > 100 μU/mL [34]), and triglyceride levels at concentrations that were consistent with metabolic syndrome (fasting triglyceride > 79.7 mg/dL [35]) (Supplementary Table S7). Since the correlation that is found in Figure 5b appeared to be mainly driven by the two animals with very high HOMA-IR levels, a Spearman correlation test was further applied, which confirmed the significant positive correlation between the two measurements (*p* = 0.022, ρ = 0.63).

Among 46 metabolites that were profiled from infant plasma, TCA cycle metabolites as well as glucose did not show statistically significant differences between the groups (Supplementary Table S8). Although the plasma 17 cytokines and chemokines (Supplementary Figure S2), as well as insulin that were tested herein did not show a significant group difference, an inflammation marker, 2-hydroxyisovalerate, was significantly higher in the plasma of infants from Obese mothers (*p* = 0.097, R^2^ = 0.20; Figure 5c). No significant differences in 71 urinary metabolites were found between the groups (Supplementary Table S9).

Infant plasma samples were collected under four different conditions at PD110 to assess cortisol metabolism. While no significant group difference was found at either time point (Figure 5d), infants from the Obese group showed greater suppression in response to Dex compared to those from the Lean group with statistical significance (*p* < 0.001, d = 1.2; Figure 5e).

### 3.5. mTOR Pathway of the Prefrontal Cortex in Infants Born to Obese Mothers May Be Elevated

**Figure 6:**
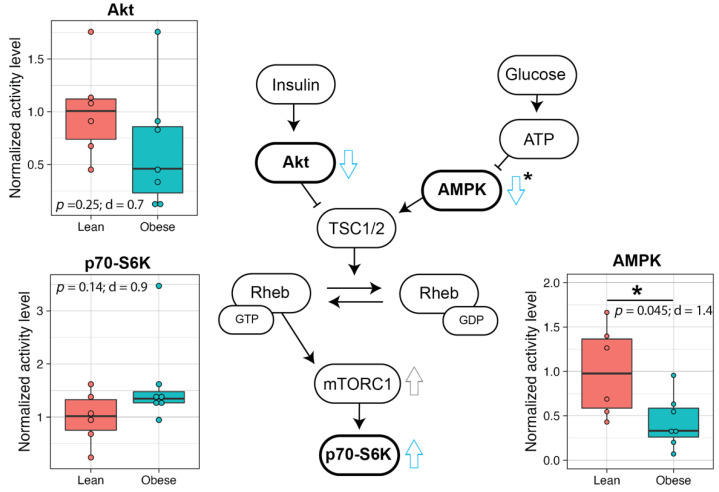
Infants that were born to Obese mothers showed elevated levels of mTOR phosphorylated proteins in the prefrontal cortex. The overall scheme of the mTOR pathway with normalized phosphorylation levels (phospho-protein/total-protein) of Akt, AMPK, and p70-S6K that were measured in the prefrontal cortex. *t*-tests were used to test for group differences and statistical significance between groups is denoted with an asterisk. The data are expressed as the mean ± SE, and the red and blue bars correspond to the Lean and Obese groups, respectively. The trends that were found in the brains of infants from the Obese group compared to the Lean group are denoted with blue open arrows. Statistical significance is indicated with “*”. For box plots, each dot represents data from an individual animal. Top and bottom of the boxes represent the 25th and 75th percentiles respectively; the middle line represents the median; and the top and bottom whiskers represent maximum and minimum values. Points outside of the whiskers represent outliers. The sample size is Lean = 6, Obese = 7. Abbreviations: TSC1/2, Tuberous sclerosis proteins 1 and 2; Rheb, Ras homolog enriched in brain; Eff, effect size.

As the mTOR pathway plays an important role in neurodevelopment, activities of enzymes in the brain mTOR pathway were assessed by measuring phosphorylation levels of AMPK, Akt, and p70-S6K by Western blot (Supplementary Figure S3). A significantly lower level of phosphorylated AMPK was found in the prefrontal cortex of infants from Obese mothers compared to infants from Lean mothers with a large effect size (*p* = 0.045, d = 1.4; Figure 6). The level of phosphorylated Akt trended lower with a medium effect size (*p* = 0.25, d = 0.7) and that of p70-S6K, which is downstream of mTORC1, tended higher with a large effect size (*p* = 0.14, d = 0.9) in the prefrontal cortex of infants that were born to Obese mothers. No significant differences in the phosphorylation levels of these enzymes were found in other regions of the brain (Supplementary Figure S4).

### 3.6. High Maternal Adiposity Led to Differences in Brain Function of Offspring

**Figure 7:**
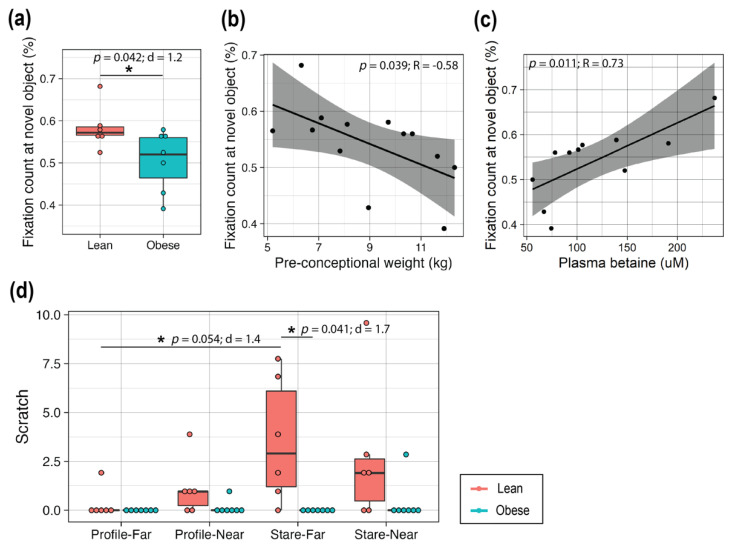
Assessment of brain function in offspring. (**a**) Proportional fixation count (%) at the novel object in the VPC test that was applied to infants at PD30. (**b**) Correlation between the pre-conceptional weight and the proportional fixation count at a novel object. (**c**) Correlation between the maternal plasma betaine level at GD90 and the proportional fixation count at a novel object. In (**b**,**c**), the black line represents the Pearson correlation regression line, and the gray area represents the 95% confidence interval. (**d**) Results of the HI test at PD110. Profile-Far (a technician presented the profile face from ~1 m away from the infant in a cage); Profile-Near (profile face presented from 0.3 m away); Stare-Far (a technician stared into the eyes of the infant from ~1 m away); Stare-Near (stared from 0.3 m away). *t*-tests were used to test for group differences in (**a**,**c**). For box plots (a,d), each dot represents data from an individual animal. Top and bottom of the boxes represent the 25th and 75th percentiles respectively; the middle line represents the median; and the top and bottom whiskers represent maximum and minimum values. Points outside of the whiskers represent outliers. Statistical significance is indicated with “*”. The data are expressed as the mean ± SE, and the red and blue bars correspond to the Lean and Obese groups, respectively. Sample size: Lean = 6, Obese = 7. Abbreviations: Eff, effect size; d or R, effect size measurements; VPC, Visual Paired-Comparison; HI test, Human Intruder test; PD, postnatal day.

To assess neurodevelopment, specifically recognition memory development, a VPC test was conducted, and novelty preference of infants on post-conception day 200 ± 3 days (~1 month of age) was compared between the groups. Typically, developed animals prefer to observe a novel object over a familiar one for longer duration with a higher frequency [21,22]. Here, the proportional fixation count at novel objects at PD30 was significantly lower with a large effect size in infants from mothers in the Obese group compared to the Lean group (*p* = 0.042, d = 1.2; Figure 7a). Indeed, maternal pre-conceptional weight and proportional fixation count at novel objects had a significant negative correlation (*p* = 0.039, R = −0.58; Figure 7b). Also, interestingly, the maternal plasma betaine levels at GD90, 120 and 150 showed a strong positive correlation with proportional fixation count at novel objects (*p* = 0.011, R = 0.73, Figure 7c).

The HI test was conducted on infants at PD110. This test comprised of four 1-min trials with graded levels of stress, and the frequency of scratch within each trial was used as the measurement of anxiety [23] (Figure 7d). The infants of mothers in the Lean group showed the appropriate response, with a higher frequency of scratching in Profile-Near than Profile-Far (*p* = 0.26, d = 0.79), as well as in Stare-Far than Profile-Far (*p* = 0.054, d = 1.4). However, infants of mothers in the Obese group did not show distinct reactions between the different conditions. In addition, infants of mothers in the Obese group showed significantly lower frequencies in the Stare-Far condition compared to those of the Lean group (*p* = 0.041, d = 1.7).

## 4. Discussion

Maternal gestational obesity has been suggested to be a risk factor for aberrant brain development in offspring [3]. A number of studies have investigated the impact of an HFD on placental function [36,37], fetal metabolism [36,38], as well as the cognitive and behavioral outcomes of offspring [39,40]. However, these study designs are not appropriate to understand the impact of maternal adiposity itself, as diet is a large confounding factor that can predispose offspring to a metabolically unfavorable environment [38]. To separate the impact of diet and investigate the impact of high maternal gestational adiposity on the cognitive development of offspring, we used rhesus macaques that naturally developed and maintained obesity over time, even though they consumed the same diet as the Lean mothers. In this study, only male infants were included, as ASD is more prevalent in males than females [41] and the effects of maternal obesity on cognition and behavior are observed more often in male infants [42].

In normal pregnancies, maternal tissues become increasingly insensitive to insulin [10]. This “insulin resistance” occurs in healthy pregnancies to limit the amount of glucose that is metabolically utilized by mothers and ensures a sufficient energy supply to the growing fetus [10]. In this study, the Obese mothers showed a progressive increase in insulin resistance that was measured by the HOMA-IR over the course of pregnancy, despite having a lower GWG rate compared to the Lean mothers. Indeed, the average GWG at GD150 of Obese mothers was almost four-times higher than that of the Lean controls. Unlike HFD-induced obese animal studies and human studies where pregnant mothers with overweight/obese BMI may have a diet that is rich in calories, all the animals in our study were fed the same amount of the same standard diet. Therefore, this result suggests that pre-pregnant adiposity itself can have effects on pregnancy outcomes that are independent of excessively high GWG. This is important because GWG has been associated with aberrant pregnancy outcomes, and as such the Institute of Medicine guidelines that were set in 2009 recommends that mothers with obesity control GWG [17,43,44]. It is interesting that controlling GWG in pregnant women with obesity resulted in mixed efficacy in preventing health complications in mothers and offspring, with some studies suggesting that adverse pregnancy outcomes may be independent of GWG [44]. Also, insulin resistance in subjects with obesity is primarily derived by factors from excessive fat [10]. Thus, pre-conceptional adiposity may help predict pregnancy outcome better than GWG. 

HOMA-IR was positively correlated with EPV and infant early weights, which is concordant with a previous study in human pregnancies [45]. These results suggest that insulin resistance impacts placental size and function resulting in more energy to the fetus and increased fetal growth despite a considerably lower GWG (this has also been shown in humans [26,30]). Interestingly, mothers in the Obese group showed larger variability in their HOMA-IR profile compared to the Lean group. This is likely because the impact of obesity in insulin resistance is not the same for each individual. Indeed, one study showed high heterogeneity in the metabolic characteristics of obese monkeys as they progressed toward Type 2 diabetes mellitus even if they showed a similar percentage of body fat [34].

One consequence of obesity could be changes in the redox balance between the maternal-placental-fetal unit [46], leading to higher oxidative stress. As expected, Obese mothers showed higher levels of an inflammatory marker (hs-CRP) throughout pregnancy compared to the Lean mothers, suggesting a chronic state of inflammation, although other cytokines did not show a significant group difference. In the Obese group, maternal plasma betaine and DMG were lower, whereas urinary betaine and DMG were higher compared with the Lean group. These differences could suggest that betaine-homocysteine S-methyltransferase (BHMT) activity in the liver and/or kidney was higher in the Obese group. Similar trends have been reported in the plasma and urine from patients with diabetes [47], and the plasma of diet-induced obese baboons [48]. Additionally, the higher expression of hepatic BHMT was reported for diet-induced obese mice [49]. Considering that high homocysteine, shown to be induced by obesity [48], is associated with complications in pregnancy such as an enhanced risk of placenta-mediated pregnancy complications, including early pregnancy loss, fetal growth restriction, preeclampsia, and placental abruption [50], the elevation in BHMT expression may be an adaptive mechanism to cope with accumulated homocysteine. Since the sample size of placental samples was very small, we must consider the results as exploratory. However, we observed trends in the metabolites in both the methionine cycle and transsulfuration pathway in placental tissue that included lower levels of choline, betaine, and glutathione in mothers from the Obese group compared to the Lean group. Since glutathione is the main anti-oxidant that is found in cells [51], fetuses of the Obese group may have had less protection against oxidative stress in utero. This is supported by the increased level of plasma 2-hydroxyisovalerate in infants from the Obese group throughout the experiment. Of interest, 2-hydroxyisovalerate was previously shown to be significantly elevated in amniotic fluid and fetal brain in response to intrauterine lipopolysaccharide injection [52] and has been observed to be elevated in children with obesity [53,54].

Another metabolic alteration that was found in our study infants was the potential dysfunction of the hypothalamus-pituitary-adrenal (HPA) axis. As no group difference was found in the morning or afternoon cortisol levels, as well as in response to an ACTH injection, maternal obesity did not appear to affect the ability of the infant’s adrenal gland to synthesize cortisol, which is concordant with a previous study that evaluated the impact of maternal adiposity and GWG on rhesus macaque infants [17]. However, infants in the Obese group showed greater suppression of plasma cortisol in response to Dex, suggesting that they had enhanced negative glucocorticoid feedback sensitivity. Importantly, higher pre-pregnancy BCS has been shown to be associated with lower cortisol levels after Dex injection in rhesus macaques [17], and Dex super-suppressors have been identified in rhesus macaques that are exposed to chronic social stress [55], as well as with patients with posttraumatic stress disorder [56] and depression [13].

To assess the impact of obesity on offspring neurodevelopment, we performed the VPC test, which has been utilized in a number of studies to discern the impact of perinatal risk conditions on brain development [22]. In this study, we found that infants from Obese mothers showed significant reductions in novelty preference compared to infants from Lean mothers. The similar association between the maternal high pre-pregnant adiposity and reduced interest in novel stimuli in offspring was reported in our larger study of rhesus macaques (N = 173) [17].

The maintenance of homeostasis of synaptic protein synthesis and degradation by autophagy is critical for learning, memory formation, as well as neurodevelopment [12]. Importantly, one of the mTOR proteins, AMPK, plays an essential role in synaptic plasticity, and therefore, its activity in the brain was studied to explore whether this may explain the differences in VPC test performance. Under energy-sufficiency, AMPK activity is lowered leading to an elevation of mTORC1 via the Tuberous Sclerosis Complex, and autophagy-initiating kinase (Ulk1) activation is prevented by being phosphorylated at Ser 757 by mTORC1 [9]. In this study, we found that the infants of Obese mothers had significantly lower levels of AMPK activity and a higher activity of p70-S6K in the prefrontal cortex. In a previous study, it was shown that postmortem brains of individuals with ASD had an over-activation of the mTOR pathway together with a deficiency in autophagy, suggesting dysregulation in synaptic plasticity during childhood and adolescence [10]. Although mTORC1 regulates several transcriptional functions, based on this previous work and our observations here, we suggest that maternal obesity impacts infant brain mTORC1 activity resulting in an inhibition of autophagy of synaptic proteins. Thus, the difference in cognitive development that we found in the infants from mothers in the Obese group may be explained by an upregulation of the brain mTORC1 pathway.

We found that maternal plasma betaine at GD90, 120, and 150 showed a strong positive correlation with the offspring proportional fixation count at a novel object. Betaine is associated with DNA methylation, which regulates brain development in utero and in early perinatal life [40,57]. Previous work has shown that male offspring that were exposed to a perinatal maternal HFD challenge had a reduction in the whole-genome DNA methylation in the prefrontal cortex and deficits in cognitive functions that were associated with the prefrontal cortex [40,57]. Interestingly, these adverse outcomes were attenuated by postnatal methyl-donor supplementation [39,40,58]. Considering that plasma concentrations of methyl donors from pregnant women with obesity and their fetuses have been found to be lower than those from lean pregnant women [29], and perinatal folic acid supplementation was associated with the lowered risk for ASD [59], changes in methylation may be one mechanism to explain ASD etiology.

Lastly, the HI test was utilized to assess anxious behavior of infants in response to graded conditions of stress and challenge at the age of PD110. Infants that were born to the Lean mothers showed appropriate responses that were proportional to the level of challenge, whereas those that were born to Obese mothers showed little difference. Anxious temperament in rhesus macaques has been previously associated with glucose metabolic rate in anterior temporal lobe clusters (e.g., amygdala, hippocampus) [60], which could potentially be impacted by maternal obesity.

## 5. Conclusions

Limitations of the current study include a small sample size, the unavailability of sufficient placental samples from all the mothers, and an initial focus on male offspring only. Future studies on a larger cohort as well as the inclusion of female offspring would be of great interest. Nonetheless, our results provide intriguing insights into how maternal obesity may alter maternal, placental, fetal, and infant brain metabolism, and how it may be linked with the neurodevelopmental outcomes of offspring. Specifically, our rhesus macaque study ruled out the impact of diet and allowed assessing the effect of high pre-conceptional adiposity on the pregnancy outcomes in a more comprehensive manner, which cannot be done in human studies. Furthermore, infants that were born to mothers with high pre-conceptional adiposity had impacts on metabolic, cognitive, and behavioral development, which were associated with certain maternal parameters during pregnancy. Many of our results align with previous findings from animal and human studies, and our work was able to capture changes in pathways that might reflect vulnerability in infant brain development that is induced by maternal obesity. 

## Data Availability

Data are available in the publicly accessible repository Dryad at https://doi.org/10.5061/dryad.mpg4f4r31 (accessed on 16 August 2022).

## Supplementary Materials

The following supporting information can be downloaded at: https://www.mdpi.com/article/10.3390/metabo12080764/s1, Figure S1: Maternal plasma cytokine levels illustrating no significant group differences.; Figure S2: Infant plasma cytokine levels show no significant group differences.; Figure S3: The representative Western blotting raw images.; Figure S4: Activity of mTOR proteins in amygdala, hippocampus, and hypothalamus of infants that were born to mothers in the Obese group vs. Lean group.; Table S1: Summary of animals that were used for each of biological sample that was used in this study.; Table S2: Summary of sample size that was used in the analyses.; Table S3: Summary of coefficient of variation (%) of HOMA-IR.; Table S4: Concentrations of metabolites in maternal plasma.; Table S5: Concentrations of metabolites in maternal urine.; Table S6: Concentrations of metabolites in placenta.; Table S7: Metabolic status of the two Obese mothers who showed high HOMA-IR and large birthweight of infants in Figure 5b.; Table S8: Concentrations of metabolites in infant plasma.; Table S9: Concentrations of metabolites in infant urine.

## Appendix A

### Appendix A.1. Feeding and Rearing of Animals

Pregnant females were relocated to the project room on approximately GD70, and approximately two weeks after relocation, compatible mothers were paired during daytime hours. The mothers were allowed to deliver naturally, and cesarean deliveries were performed for postdate pregnancies of ~GD175 or as recommended by veterinarians. A foster mother was provided if the biological mother did not accept her infant following caesarean (2 animals each for both groups). Mother-infant dyads were housed indoors and paired with their compatible mother-infant dyad during daytime hours.

### Appendix A.2. Metabolite Extraction and Insulin, Cytokine, and Cortisol Measurement

Insulin and cytokine levels were analyzed on a Bio-Plex 100 (Bio-Rad, Hercules, CA, USA). A minimum of fifty beads per region were collected for each sample, and data was analyzed with Bio-Plex Manager software using a 5-point standard curve with immune marker quantities extrapolated based on a standard curve.

For cortisol measurement, blood samples were collected, processed, and stored as described earlier, and cortisol concentrations were obtained using a chemiluminescent assay on an ADVIA Centaur CP platform (Siemens Healthcare Diagnostics, Tarrytown, NY, USA).

### Appendix A.3. Protein Analysis

Total protein was quantified by DC Protein Assay kit (Bio-Rad).

The primary antibodies were all prepared at 1:1000 dilution in 5% *w*/*v* bovine serum albumin in Tris Buffered Saline with 0.1% *v*/*v* Tween 20 (TBST). The secondary antibody was prepared at 1:2000 dilution in 5% *w*/*v* non-fat dry milk in TBST.

### Appendix A.4. Statistics

All statistical analyses were conducted in R (version 3.6.1).

Levene’s test as well as the Shapiro–Wilk test of normality were applied to ensure plasma and urine metabolomics data met the assumptions to apply parametric tests.

The covariates (maternal age, pre-gestational weight and BCS, maternal weight, and GWG at sample collection) were included in the model if the fit of the model based on Akaike information criterion values were improved by inclusion, and when the majority of metabolites showed significant *p*-values when compared with an unadjusted model. As a result, the regression model was adjusted for body weight for maternal plasma, GWG for maternal urine, and no covariates were added for the rest of samples. As the body weight and GWG were critical measurements that distinguished the groups, the covariates were centered by subtracting the mean value of the assigned group at each time point from each measurement value in order to only adjust for the variability within the group.

One sample (animal ID = 1215218 from the Obese group) was removed from the generation of Figure 1a as there were technical errors in the measurement of insulin levels. Although the statistics were applied to samples that were collected from mothers who had successful deliveries of male infants, due to the extremely small sample size, the metabolomics analysis on the placental samples also included two samples that were collected from obese mothers who had fetal death (Supplementary Table S1).

A theoretical sample size for this study was chosen based on CNPRC primate infant bio-behavioral assessment records, which revealed that 50% of infants from mothers that weighed more had greater emotionality and signs of poor adaptation compared to infants of mothers with a healthy body condition. Thus, the study was powered to observe differences in behavior, with a sample size of 8 monkeys per group providing a power of 80–93% to detect a difference in the rate of change in duration of looking over a period of 6 months.
