# Supplementary material for: Impact of Maternal Obesity on the Gestational Metabolome and Infant Metabolome, Brain, and Behavioral Development in Rhesus Macaques
Source: Metabolites. 2022 Aug 19;12(8):764. doi: 10.3390/metabo12080764 (PMC9415340; doi:10.3390/metabo12080764)
Supplement: Supplementary file 1 [file metabolites-12-00764-s001.zip › metabolites-1848833-supplementary.pdf]

Supplementary Materials

Impact of Maternal Obesity on the Gestational Metabolome and Infant Metabolome, Brain, and Behavioral Development in Rhesus Macaques

Yu Hasegawa, Zhichao Zhang, Ameer Y. Taha, John P. Capitanio, Melissa D. Bauman, Mari S. Golub, Judy Van de Water, Catherine A. VandeVoort, Cheryl K. Walker, Carolyn M. Slupsky

- **Supplementary Figures:**
  - Supplementary Figure S1. Maternal plasma cytokine levels illustrating no significant group differences.
  - Supplementary Figure S2. Infant plasma cytokine levels show no significant group differences.
  - Supplementary Figure S3. The representative Western blotting raw images.
  - Supplementary Figure S4. Activity of mTOR proteins in amygdala, hippocampus, and hypothalamus (mean ± SE) of infants born to mothers in the Obese group vs Lean group.

- **Supplementary Tables:**
  - Supplementary Table S1. Summary of biological samples used in this study.
  - Supplementary Table S2. Summary of sample size used in the analyses.
  - Supplementary Table S3. Summary of coefficient of variation (%) of HOMA-IR.

- Supplementary Table S4. Mean concentrations of metabolites in maternal plasma (μM).
- Supplementary Table S5. Mean concentrations of metabolites in maternal urine (μM).
- Supplementary Table S6. Mean concentrations of metabolites in placenta (nmol/g).
- Supplementary Table S7. Metabolic status of the two Obese mothers who showed high HOMA-IR and large birthweight of infants in Figure 4b.
- Supplementary Table S8. Mean concentrations of metabolites in infant plasma (μM).
- Supplementary Table S9. Mean concentrations of metabolites in infant urine (μM).

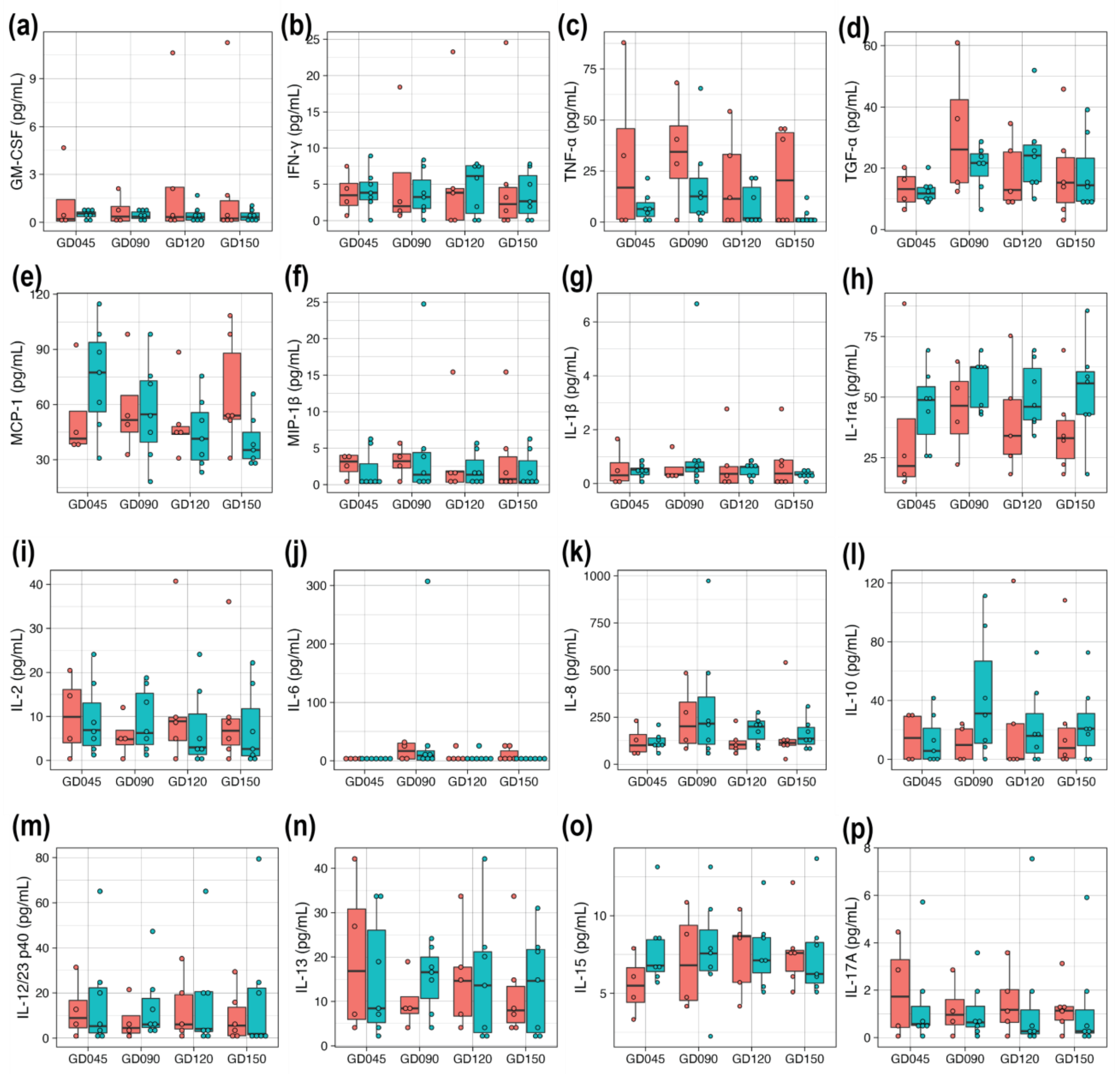

Supplementary Figure S1. Matenral plasma cytokine levels illustrating no significant group differences. Bar plots represent (a) GM-CSF, (b) IFN-γ, (c) TNF-α, (d) TGF-α, (e) MCP-1, (f) MIP-1β, (g) IL-1β, (h) IL-1ra, (i) IL-2, (j) IL-6, (k) IL-8, (l) IL-10, (m) IL-12/23 p40, (n) IL-13, (o) IL-15, and (p) IL-17A. Each dot represents data from an individual animal. Top and bottom of the boxes represent the 25th and 75th percentiles respectively; the middle line represents the median; and the top and bottom whiskers represent maximum and minimum values. Points outside of the whiskers represent outliers. The red and blue correspond to the Lean and Obese groups respectively.

Abbreviations: GM-CSF, granulocyte-macrophage colony-stimulating factor; IFN- γ, interferon γ; TNF-α, tumor necrosis factor-α; TGF-α, transforming growth factor-α; MCP-1, monocyte chemoattractant protein-1; MIP-1β, macrophage inflammatory protein-1β; IL, interleukin.

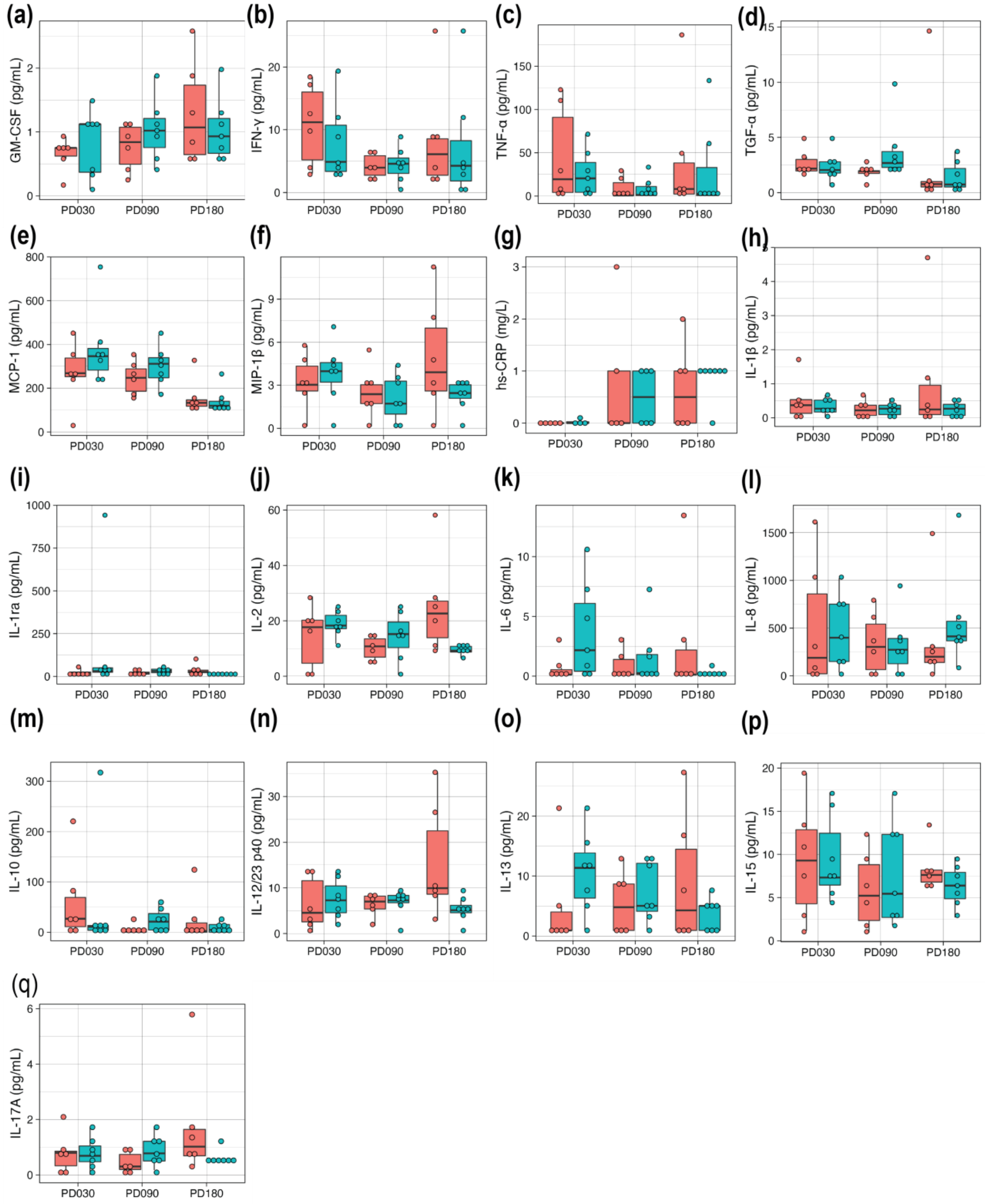

Supplementary Figure S2. Infant plasma cytokine levels show no significant group differences. Bar plots represent (a) GM-CSF, (b) IFN-γ, (c) TNF-α, (d) TGF-α, (e) MCP-1, (f) MIP-1β, (g) hs-CRP, (h) IL-1β, (i) IL-1ra, (j) IL-2, (k) IL-6, (l) IL-8, (m) IL-10, (n) IL-12/23 p40, (o) IL-13, (p) IL-15, and (q) IL-17A. Each dot represents data from an individual animal. Top and bottom of the

boxes represent the 25th and 75th percentiles respectively; the middle line represents the median; and the top and bottom whiskers represent maximum and minimum values. Points outside of the whiskers represent outliers. The red and blue correspond to the Lean and Obese groups respectively.

Abbreviations: GM-CSF, granulocyte-macrophage colony-stimulating factor; IFN- γ, interferon γ; TNF-α, tumor necrosis factor-α; TGF-α, transforming growth factor-α; MCP-1, monocyte chemoattractant protein-1; MIP-1β, macrophage inflammatory protein-1β; hs-CRP, high-sensitivity C-reactive protein; IL, interleukin.

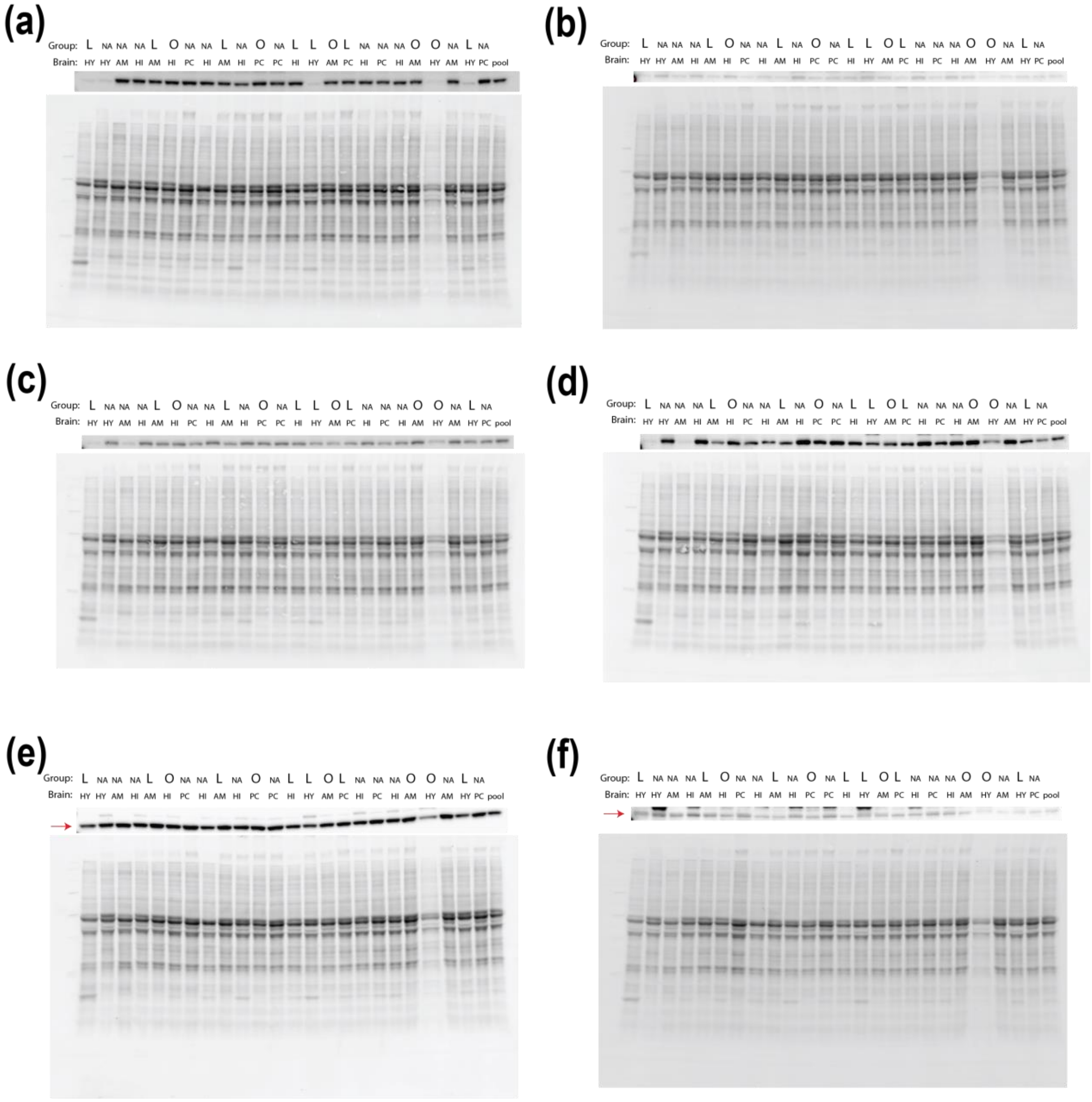

Supplementary Figure S3. Representative Western blot raw images. The band image (above) and the stain-free image showing the total protein (below) are shown for (a) total Akt, (b) p-Akt, (c) total AMPK, (d) p-AMPK, (e) total p70-S6K, and (f) p-p70-S6K. The samples from all 4 brain regions were randomized and run with the pooled sample (indicated "pool" in the image) used to normalize between different runs. Samples from different regions of brain were noted with the abbreviation of the brain regions (AM, amygdala; HI, hippocampus; HY, hypothalamus; PC, prefrontal cortex). The samples from Lean group are indicated as "L" and those from Obese group are indicated with "O", and those that were not used in this study are indicated with "NA".

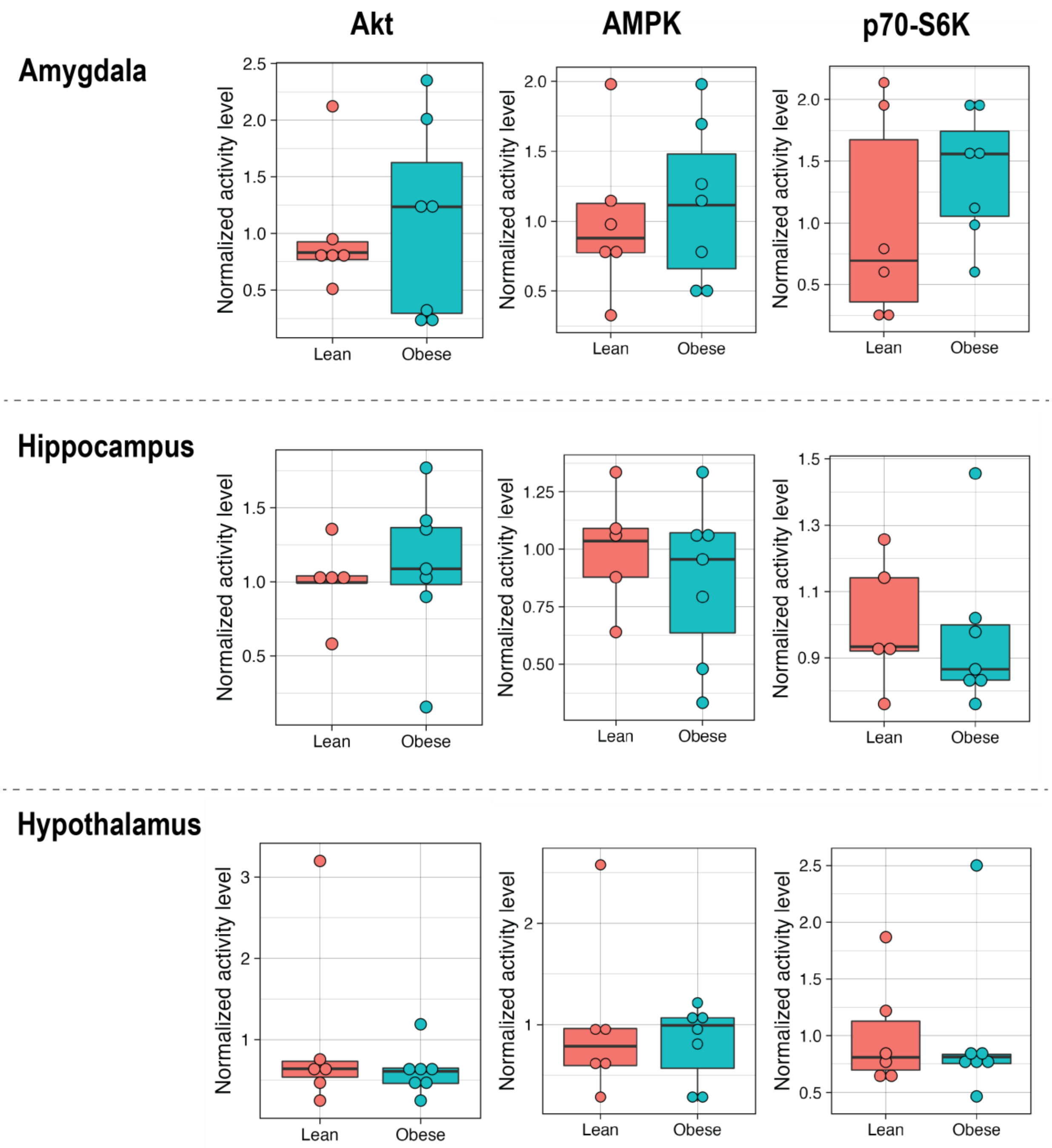

Supplementary Figure S4. Activity of mTOR proteins in amygdala, hippocampus, and hypothalamus of infants born to mothers in the Obese group vs Lean group. The red and blue bars correspond to infants of mothers in the Lean and Obese groups respectively. Each dot represents data from an individual animal. Top and bottom of the boxes represent the 25th and 75th percentiles respectively; the middle line represents the median; and the top and bottom whiskers represent maximum and minimum values. Points outside of the whiskers represent outliers. Sample size: Lean=6 for amygdala and hypothalamus, and n=5 for hippocampus; Obese=7 for all brain regions.

Supplementary Table S1. Summary of biological sample used in this study. Maternal samples were collected at gestational day (GD) 45, 90, 120, and 150. Placental samples were collected at GD150. Infant samples were collected at postnatal day (PD) 30, 90, 110, and 180. The sample size of the biological samples was not balanced due to fetal deaths for unknown reasons, misidentification of female offspring, technical issues in collecting enough sample volume for analysis, or recruitment of additional animals into the study in the middle of pregnancy to account for the sample loss.

	Mother ID	Infant ID	Foster Mother ID	Mode of delivery	Gestational length (days)	Maternal sample									Infant sample								
						Plasma				Urine				Placenta	Plasma				Urine	Amyg-dala	Hippo-campus	Hypo-thalamus	Pre-frontal cortex
						GD45	GD90	GD120	GD150	GD45	GD90	GD120	GD150	GD150	PD30	PD90	PD110	PD180	PD180	PD180	PD180	PD180	PD180
**Lean**	1146126	NA	NA	NA	NA	X	X	X		X	X	X											
	1147111	3147111	NA	Vaginal	157				X				X		X	X	X	X	X	X	X	X	X
	1145122	3145122	NA	Vaginal	163	X	X	X	X	X	X	X		X	X	X	X	X	X	X		X	X
	1142120	NA	NA	NA	NA	X				X													
	1141109	NA	NA	Vaginal	NA	X	X	X	X	X	X	X	X										
	1144110	3144110	NA	Vaginal	165			X	X			X	X		X	X	X	X	X	X	X	X	X
	1143103	NA	NA	NA	NA	X	X			X	X												
	1242201	3242201	NA	Vaginal	167	X	X	X	X	X	X	X	X	X	X	X	X	X	X	X	X	X	X
	1241217	3241217	2241217	C-section	150	X	X	X	X	X	X	X	X		X	X	X	X	X	X	X	X	X
	1243225	3243225	2243225	C-section	165	X	X	X	X	X	X	X			X	X	X	X	X	X	X	X	X
	1244124	NA	NA	Vaginal	NA	X	X	X	X	X	X	X											
**Obese**	1113127	NA	NA	NA	NA		X	X	X		X	X	X										
	1111101	NA	NA	Vaginal	NA	X	X	X	X		X	X	X										
	1114102	3114102	NA	Vaginal	176	X	X	X	X	X	X	X	X		X	X	X	X	X	X	X	X	X
	1115104	NA	NA	NA	NA	X	X	X	X	X	X	X		X									
	1112102	3112102	NA	Vaginal	170	X	X	X	X	X	X	X	X		X	X	X	X	X	X	X	X	X
	1212115	3212115	NA	Vaginal	168	X	X	X	X	X	X	X	X		X	X	X	X	X	X	X	X	X
	1213116	NA	NA	NA	NA	X	X	X	X	X	X	X	X	X									
	1216119	3216119	NA	Vaginal	158	X	X	X	X	X	X	X	X		X	X	X	X	X	X	X	X	X
	1214217	3214217	2214217	C-section	162	X	X	X	X	X	X	X	X	X	X	X	X	X	X	X	X	X	X
	1215218	3215218	2215218	C-section	167	X	X	X	X	X	X	X	X		X	X	X	X	X	X	X	X	X
	1211112	3211112	NA	Vaginal	161	X	X	X	X	X	X	X	X	X	X	X	X	X	X	X	X	X	X

Supplementary Table S2. Summary of sample size used in the analyses.

	Mother								Infant							
	**GD45**		**GD90**		**GD120**		**GD150**		**PD30**		**PD90**		**PD110**		**PD180**	
	**Lean**	**Obese**	**Lean**	**Obese**	**Lean**	**Obese**	**Lean**	**Obese**	**Lean**	**Obese**	**Lean**	**Obese**	**Lean**	**Obese**	**Lean**	**Obese**
Plasma	4	7	4	7	5	7	6	7	6	7	6	7	6	7	6	7
Urine	4	7	4	7	5	7	4	7							6	7
Placenta							2	4								
Amygdala															6	7
Hippo-campus															5	7
Prefrontal cortex															6	7
Hypo-thalamus															6	7

Supplementary Table S3. Summary of coefficient of variation (%) of HOMA-IR.

Group	GD45	GD90	GD120	GD150
Lean	61	76	44	78
Obese	56	100	123	106

Supplementary Table S4. Mean concentrations of metabolites in maternal plasma (µM). FDR correction was applied on p-values of metabolites involved in TCA cycle or one-carbon metabolism pathway. Concentrations are expressed as mean and standard deviation (SD). Effect size was calculated for metabolites with FDR corrected p<0.1.

	LC								OC								p-value (adjusted p-value)	Effect size (R2)
	GD45		GD90		GD120		GD150		GD45		GD90		GD120		GD150			
	Mean	SD	Mean	SD	Mean	SD	Mean	SD	Mean	SD	Mean	SD	Mean	SD	Mean	SD		
2-Hydroxybutyrate	12.1	6.0	13.3	6.6	9.8	4.8	11.2	4.2	17.9	11.3	8.5	4.8	10.1	4.0	9.5	6.2	0.71	
2-Hydroxyisovalerate	4.0	1.2	3.1	1.3	3.7	1.3	3.8	1.0	6.6	5.1	3.5	1.3	4.2	2.1	4.6	2.7	0.44	
2-Oxoglutarate	9.6	0.8	6.5	1.0	7.9	1.5	8.3	1.3	11.4	1.8	9.4	3.6	10.3	2.3	10.2	3.2	0.085 (0.085)	0.23 (medium)
2-Oxoisocaproate	9.9	3.0	7.2	1.8	8.1	2.7	7.1	1.8	8.2	1.0	7.3	1.9	7.6	1.9	6.7	1.1	0.90	
3-Hydroxybutyrate	92.8	41.2	176.8	115.0	79.5	43.7	53.2	22.5	161.5	166.2	91.8	73.6	68.2	34.5	67.8	67.7	0.74	
3-Hydroxyisobutyrate	26.5	4.5	27.5	6.3	26.1	3.7	22.2	3.9	20.4	2.2	20.9	3.7	20.6	3.3	20.1	2.8	0.013	
3-Methylhistidine	39.2	11.7	44.9	6.6	53.3	14.5	56.7	15.1	38.0	10.7	65.2	8.4	68.2	7.0	72.3	6.6	0.035	
Acetate	68.7	21.5	105.9	26.6	96.8	25.3	112.1	50.8	115.6	64.2	128.8	62.2	89.8	44.1	112.3	43.9	0.59	
Acetoacetate	58.2	27.5	122.0	69.7	50.4	30.7	44.9	13.5	120.0	126.3	67.1	47.6	45.5	18.7	59.7	57.2	0.94	
Acetone	13.9	4.5	17.1	9.8	12.0	4.9	12.2	3.0	20.8	17.7	12.1	6.5	11.7	5.8	16.1	19.1	0.74	
Alanine	221.5	25.0	146.2	55.8	176.5	44.1	167.6	10.2	218.2	34.2	182.1	19.1	192.5	44.9	183.4	9.2	0.10	
Arginine	51.3	16.1	42.4	1.2	43.4	11.7	42.5	10.2	55.1	5.3	48.5	12.1	47.3	8.9	45.7	9.5	0.21	
Asparagine	32.0	5.5	18.5	4.5	25.4	3.0	24.9	3.8	27.4	3.1	26.6	6.4	26.2	4.2	25.9	3.6	0.33	
Betaine	66.2	24.7	145.7	63.3	154.6	67.1	162.4	64.6	68.1	16.6	100.9	49.6	126.4	56.8	153.9	76.2	0.53 (0.79)	
Butyrate	7.2	2.4	6.2	1.0	6.1	2.0	6.1	1.1	6.8	1.0	5.9	1.1	5.6	1.4	5.2	1.2	0.38	
Carnitine	28.5	7.1	26.0	14.1	25.8	6.1	22.7	7.7	22.8	9.2	22.1	6.1	23.6	4.6	21.3	7.8	0.56	
Choline	8.9	6.7	5.5	1.7	5.2	1.5	6.7	4.7	6.2	4.1	8.4	6.1	7.2	5.8	5.8	1.3	0.82 (0.82)	
Creatine	77.2	21.7	55.2	18.0	57.3	5.8	56.6	11.2	45.5	18.8	60.5	12.5	62.1	16.0	62.1	24.8	0.55	
Creatinine	50.7	6.6	49.7	8.8	53.0	14.2	47.9	9.5	50.2	6.0	48.5	4.0	51.0	5.2	50.1	2.6	0.62	
Ethanol	23.1	14.3	19.5	20.9	32.1	25.0	61.2	85.6	34.1	29.6	23.3	15.5	15.6	11.3	18.7	5.8	0.64	
Formate	106.3	7.2	99.3	18.8	91.8	14.5	112.1	16.3	92.6	21.5	113.1	18.9	114.8	21.6	119.5	22.5	0.54	

Glucose	3592.5	336.4	3388.5	561.7	3665.3	1176.8	3260.9	451.4	4504.3	1648.4	3277.3	242.2	3692.1	1005.5	3488.7	290.1	0.32	
Glutamate	31.5	10.0	46.5	16.9	31.1	11.3	36.2	14.8	49.4	26.2	46.6	16.2	32.3	9.1	39.5	9.0	0.41	
Glutamine	331.0	40.4	266.5	19.9	311.1	36.1	324.7	37.0	370.4	36.7	329.1	22.8	319.6	49.1	328.3	26.2	0.11	
Glycerol	559.7	75.4	452.3	35.0	500.2	60.9	542.0	128.8	550.2	83.0	497.0	129.4	519.4	115.2	573.7	93.7	0.43	
Glycine	253.1	43.1	221.5	38.0	217.3	14.1	210.0	17.0	283.9	44.4	239.7	28.8	232.3	29.0	227.2	20.1	0.12	
Histidine	64.0	10.3	56.4	12.8	59.4	9.5	59.5	9.8	70.7	8.5	79.9	11.2	73.5	15.5	74.3	9.4	0.0020 (0.0082)	0.49 (large)
Isoleucine	79.1	10.0	67.6	8.6	58.3	10.8	55.9	9.3	75.9	10.8	65.6	8.5	62.0	9.5	57.9	5.3	0.53	
Lactate	2944.5	2138.3	1832.9	1840.2	2719.3	2104.5	1740.6	660.3	1607.5	639.7	1401.8	502.6	2054.4	1417.0	1479.2	447.4	0.58	
Leucine	95.8	13.6	83.8	18.0	77.2	15.6	70.1	13.4	90.9	11.9	84.2	11.4	78.8	15.7	77.0	8.5	0.38	
Lysine	126.9	11.5	110.7	10.3	123.4	13.3	120.2	20.3	144.5	19.1	142.8	21.3	140.5	14.7	143.8	29.3	0.010 (0.026)	0.39 (large)
Methionine	25.9	3.4	16.3	2.7	17.1	1.6	14.8	2.1	22.3	3.6	17.8	2.9	17.1	3.0	16.0	3.7	0.92	
myo-Inositol	24.4	8.9	22.1	3.8	29.7	7.7	27.4	2.6	21.1	4.1	27.3	3.6	30.6	6.8	28.0	2.1	0.53	
N,N-Dimethylglycine	2.9	0.8	3.6	1.4	3.3	0.4	4.0	0.9	2.0	0.9	2.6	1.7	2.7	0.8	3.4	1.6	0.0020 (0.0049)	0.50 (large)
O-Acetylcarnitine	10.7	4.9	10.8	4.6	8.1	2.8	6.3	2.1	8.1	3.2	6.8	4.6	7.1	2.3	6.5	4.7	0.34	
Phenylalanine	46.1	4.4	35.1	2.7	37.4	2.2	35.8	5.9	42.0	5.0	39.8	4.7	39.6	6.6	37.6	4.7	0.41	
Proline	105.7	6.3	111.9	22.7	127.0	37.0	118.4	22.5	132.3	19.6	147.3	25.3	131.2	44.8	135.9	22.4	0.040 (0.050)	0.29 (medium)
Pyruvate	192.9	80.8	140.5	112.8	163.2	90.0	142.5	48.8	136.6	54.8	129.6	40.4	159.3	72.8	130.8	36.4	1.00	
Serine	102.9	18.5	97.3	34.9	100.3	25.3	96.9	16.6	107.8	14.4	96.2	14.2	91.4	11.3	87.4	9.8	0.38	
Succinate	2.7	0.7	1.9	0.3	2.7	1.0	2.5	0.7	3.2	0.8	2.8	0.8	3.2	0.7	2.8	0.7	0.040 (0.050)	0.29 (medium)
Taurine	105.3	34.4	80.4	13.3	94.0	17.5	83.8	25.3	79.8	15.0	83.1	14.1	91.1	17.0	89.6	11.9	0.97	
Threonine	83.2	12.0	62.9	16.9	69.7	9.9	67.0	7.3	70.5	7.3	67.9	11.8	70.4	10.1	74.7	8.1	0.63	
Tyrosine	61.7	12.0	46.3	8.9	52.0	10.1	50.7	10.5	49.5	7.1	41.5	8.1	43.5	6.7	46.7	6.3	0.070	
Urea	2722.0	681.7	3046.6	914.4	3003.9	803.9	2787.4	539.9	2217.9	505.4	2500.8	685.6	2580.8	536.9	2726.2	601.1	0.45	
Uridine	7.6	1.6	9.5	1.4	7.7	1.2	8.2	1.0	5.8	1.3	6.6	0.5	6.9	1.2	6.5	1.1	<0.001	
Valine	216.4	27.8	179.0	29.3	162.5	26.3	150.6	21.6	201.4	26.2	176.6	28.3	164.0	32.1	158.5	14.7	0.71	

Supplementary Table S5. Mean concentrations of metabolites in maternal urine (μM). FDR correction was applied on p-values of metabolites involved in TCA cycle or one-carbon metabolism pathway. Concentrations are expressed as mean and standard deviation (SD). Effect size was calculated for metabolites with FDR corrected p-values less than 0.1. Concentrations are expressed as mean and standard deviation (SD). Effect size was calculated for metabolites with FDR corrected p<0.1.

	**LC**								**OC**								**p-value (adjusted p-value)**	**Effect size (R2)**
	**GD45**		**GD90**		**GD120**		**GD150**		**GD45**		**GD90**		**GD120**		**GD150**			
	**Mean**	**SD**	**Mean**	**SD**	**Mean**	**SD**	**Mean**	**SD**	**Mean**	**SD**	**Mean**	**SD**	**Mean**	**SD**	**Mean**	**SD**		
2-Hydroxybutyrate	8	7	7	5	11	12	8	6	25	31	8	8	10	10	17	14	0.087	
2-Hydroxyisobutyrate	36	38	22	15	23	20	14	7	44	37	19	14	28	34	11	10	0.75	
2-Hydroxyisovalerate	5	5	3	2	3	3	3	1	8	6	3	3	4	3	3	2	0.25	
2-Oxoglutarate	62	51	41	32	46	26	43	5	223	180	102	99	78	65	49	48	0.12	
2-Oxoisocaproate	33	39	15	10	17	14	12	7	25	19	13	14	13	10	11	10	0.87	
3-Hydroxy-3-methylglutarate	28	27	20	15	41	50	17	16	27	18	18	16	24	23	19	15	0.47	
3-Hydroxybutyrate	22	11	43	26	51	59	45	58	825	2019	134	281	139	242	97	191	0.16	
3-Hydroxyisobutyrate	64	45	55	46	63	31	57	37	143	139	67	79	87	95	60	43	0.34	
3-Hydroxyisovalerate	85	108	63	38	71	64	53	45	89	62	58	53	93	137	71	53	0.22	
3-Indoxylsulfate	237	235	175	171	327	407	131	90	320	239	210	192	242	205	195	186	0.16	
3-Methyl-2-oxovalerate	7	6	5	5	8	6	7	6	25	30	12	22	15	20	7	7	0.24	
4-Hydroxyphenylacetate	81	76	56	31	122	126	43	9	135	132	68	58	112	156	67	34	0.33	
4-Hydroxyphenyllactate	23	20	17	10	30	27	22	13	34	22	13	11	20	10	25	21	0.47	
Acetamide	27	33	14	12	41	65	9	4	13	6	10	9	9	6	11	8	0.70	
Acetate	116	51	88	96	176	134	165	153	132	104	547	776	102	70	332	350	0.32	
Acetoacetate	29	31	64	50	84	87	331	597	725	1753	1297	2250	54	77	253	444	0.41	
Acetone	9	3	13	12	7	5	8	5	37	67	14	16	21	40	15	28	0.55	
Alanine	21	17	26	27	28	19	25	13	41	29	18	19	21	12	43	64	0.53	
Allantoin	2056	1953	1488	980	1816	2222	1282	1003	1854	1420	946	852	1787	1475	1112	815	0.68	
Arginine	131	136	69	58	95	99	70	73	190	155	54	42	80	61	57	43	0.42	

Asparagine	25	36	44	43	12	7	11	3	17	9	16	10	18	9	11	8	0.85	
Betaine	66	52	82	66	118	97	123	129	97	61	155	130	282	268	701	1236	0.029 (0.054)	0.32 (medium)
Butyrate	20	20	17	4	18	16	16	9	43	34	18	12	27	28	40	43	0.085	
Carnitine	223	229	21	29	6	7	3	3	266	352	17	18	14	19	5	4	0.32	
Citrate	319	447	217	202	280	264	76	63	1139	992	542	449	459	338	500	704	<0.010 (0.012)	0.58 (large)
Creatine	466	311	219	241	334	214	239	299	260	269	556	575	586	478	861	812	0.13	
Creatinine	5947	5861	3903	2739	6939	8578	3856	2904	7724	5594	3652	3129	5246	4563	4068	2499	0.26	
Dimethyl-sulfone	29	15	42	25	55	60	53	36	66	29	59	57	56	28	87	76	0.017	
Dimethylamine	130	129	104	81	162	197	91	65	170	123	91	70	150	149	121	69	0.15	
Ethanol	19	24	17	14	31	16	22	20	35	25	23	12	60	117	56	51	0.15	
Formate	193	176	124	69	210	190	96	33	368	301	218	198	224	160	370	474	0.043	
Fumarate	6	4	8	10	7	5	9	7	17	34	13	13	12	17	6	4	0.51	
Glucose	135	188	51	30	61	37	48	39	5876	15304	26	16	93	111	45	36	0.52	
Glucuronate	614	916	519	424	658	964	351	306	301	161	265	238	369	268	286	248	0.95	
Glutamate	139	142	72	45	110	105	62	39	123	73	72	55	87	63	77	49	0.35	
Glutamine	96	101	77	69	107	112	66	25	239	209	92	65	87	52	225	363	0.031	
Glycerol	492	421	239	35	297	142	237	53	346	71	253	100	372	104	309	96	0.37	
Glycine	62	59	37	17	97	174	40	29	113	97	35	27	59	57	120	195	0.10	
Guanidoacetate	106	108	70	60	106	90	79	48	191	209	114	114	169	123	216	248	0.054	
Hippurate	1512	1263	1146	916	1862	1648	1320	920	1869	918	1581	1783	1231	811	1719	1329	0.40	
Histidine	42	64	10	6	28	35	24	16	15	16	18	19	13	12	19	15	0.63	
Hypoxanthine	341	373	145	104	306	399	193	168	463	439	100	84	226	215	151	126	0.61	
Inosine	23	23	15	8	25	29	19	15	28	20	10	6	18	14	13	8	0.84	
Isoleucine	11	10	6	3	7	6	5	3	16	13	6	6	9	6	12	18	0.15	
Lactate	398	629	2244	4438	840	996	1465	2598	1106	1635	94	104	2985	7493	236	209	0.89	
Leucine	8	7	10	9	9	7	10	7	10	5	10	17	6	3	17	32	0.97	
Lysine	39	55	29	19	32	29	19	7	102	180	61	112	52	54	72	85	0.14	
Methionine	6	4	28	45	11	11	6	2	11	6	14	11	7	11	16	24	0.64	

myo-Inositol	81	96	27	14	63	65	29	19	98	85	31	18	48	55	45	33	0.34	
N,N-Dimethylglycine	8	6	12	13	15	9	16	17	14	8	18	15	18	9	34	30	0.054 (0.054)	0.26 (medium)
N-Carbamoyl-beta-alanine	71	67	46	32	110	169	53	36	76	55	54	57	57	55	67	52	0.34	
N-Methyl-2-pyridone-5-carboxamide	73	73	57	48	72	93	39	32	56	33	32	29	56	43	49	32	0.57	
O-Acetylcarnitine	104	185	7	7	18	24	62	110	58	88	234	439	11	11	23	29	0.35	
Pantothenate	41	41	27	18	48	60	31	26	37	25	23	19	31	24	30	21	0.43	
Phenylacetate	40	42	39	34	82	126	47	36	40	41	30	19	45	34	26	8	0.82	
Phenylalanine	31	27	25	22	51	63	24	20	44	34	16	17	30	38	28	26	0.65	
Proline	109	126	127	116	136	165	87	92	97	49	59	29	73	33	91	93	0.56	
Propylene-glycol	30	26	466	818	374	461	117	88	96	196	38	25	170	296	42	29	0.21	
Pyruvate	15	13	48	83	25	21	53	70	40	66	11	5	94	207	13	5	0.99	
Quinolinate	48	35	73	54	71	80	64	49	52	27	54	47	86	80	54	52	0.57	
Serine	43	51	29	19	54	51	34	22	80	71	37	36	36	29	68	76	0.35	
Succinate	30	32	13	8	20	17	9	4	67	60	33	44	31	40	44	65	0.061	
tau-Methylhistidine	60	62	24	17	62	93	40	56	42	59	20	17	33	28	34	40	0.93	
Taurine	3154	3016	1421	1178	1824	2040	1061	1405	2485	2174	846	1096	1224	993	1069	739	0.73	
Threonine	83	74	42	24	58	41	34	6	160	171	51	30	62	39	185	321	0.13	
Tiglylglycine	80	85	37	20	55	63	23	13	59	42	28	22	34	38	20	13	0.89	
Trimethylamine	3	3	2	1	6	9	1	1	4	4	3	3	3	3	4	2	0.12	
Trimethylamine-N-oxide	98	90	168	130	201	209	101	41	231	173	178	173	193	230	200	197	0.25	
Tyrosine	62	57	51	52	45	35	58	60	73	50	72	78	58	53	91	93	0.11	
Urea	123624	112638	102675	69146	129995	98154	84534	48247	141327	107250	85588	97877	103717	73062	118104	80359	0.59	
Valine	11	10	9	7	9	8	7	5	15	11	7	7	9	6	15	24	0.59	

Supplementary Table S6. Mean concentrations of metabolites in placenta (nmol/g). FDR correction was applied on p-values of metabolites involved in one-carbon metabolism pathway. Concentrations are expressed as mean and standard deviation (SD). Effect size was calculated for metabolites with FDR corrected p<0.1.

	LC		OC		p-value (adjusted p-value)	Effect size (Cohen's D)
	Mean	SD	Mean	SD		
3-Hydroxyisovalerate	10	9	18	2	0.29	
Acetate	927	267	1024	23	0.65	
Alanine	86	23	122	41	0.06	1.76 (very large)
Arginine	62	18	72	7	0.67	
Aspartate	219	69	246	75	0.72	
Betaine	195	47	117	64	0.038 (0.054)	1.93 (very large)
Choline	21	2	15	6	0.041(0.054)	1.95 (very large)
Creatine	57	5	64	20	0.85	
Formate	842	116	860	86	0.67	
GTP	35	6	34	25	0.46	
Glucose	567	304	725	514	0.99	
Glutamate	410	75	388	43	0.049	1.76 (very large)
Glutamine	192	9	184	32	0.30	
Glutathione	843	55	775	83	0.054 (0.054)	1.96 (very large)
Glycerol	179	13	197	38	0.31	
Glycine	141	45	135	25	0.44	
Guanidoacetate	72	32	87	10	0.33	
Isoleucine	5	9	15	12	0.76	
Lactate	762	819	1223	791	0.16	
Leucine	16	7	23	18	0.79	
Lysine	181	16	186	27	0.81	
Malonate	131	36	108	8	0.0027	7.17 (very large)
NAD	25	26	41	23	0.54	
O-Phosphocholine	44	12	30	9	0.19	
O-Phosphoethanolamine	68	13	61	13	0.66	
Propylene-glycol	66	42	311	400	0.67	
Pyruvate	44	41	67	38	0.086	1.43 (very large)
Serine	75	32	61	15	0.99	
Taurine	298	128	388	143	0.93	
Threonine	41	10	39	8	0.96	
Trimethylamine-N-oxide	224	157	134	65	0.93	
UDP-glucose	38	7	57	28	0.37	
Valine	38	13	56	33	0.85	

Supplementary Table S7. Metabolic status of the two Obese mothers who showed high HOMA-IR and large birthweight of infants in Figure 4b. Data values that correspond to the following categories are expressed as bold in the table: Hyperglycemia (fasting glucose > 100 mg/dL); hyperinsulinemia (fasting insulin > 100 μU/mL); triglyceride level that is indicative of metabolic syndrome (fasting triglyceride > 79.7 mg/dL). The asterisks represent samples that were removed due to technical errors.

Mother ID	Fasting glucose (mg/dL)				Fasting insulin (μU/mL)				Triglyceride (mg/dL)			
	GD45	GD90	GD120	GD150	GD45	GD90	GD120	GD150	GD45	GD90	GD120	GD150
1215218	70	57	104	62	261	*	*	480	38	100	127	87
1214217	147	60	60	64	2	509	720	840	127	122	111	228

Supplementary Table S8. Mean concentrations of metabolites in infant plasma (μM). FDR correction was applied on p-values of metabolites involved in TCA cycle. Concentrations are expressed as mean and standard deviation (SD). Since none of the metabolites had adjusted p-values < 0.05, effect size was not calculated.

	LC								OC								p-value (adjusted p-value)
	PD30		PD90		PD110		PD180		PD30		PD90		PD110		PD180		
	Mean	SD	Mean	SD	Mean	SD	Mean	SD	Mean	SD	Mean	SD	Mean	SD	Mean	SD	
2-Hydroxybutyrate	21	2	21	3	12	3	21	8	19	4	19	5	11	6	31	28	0.69
2-Hydroxyisovalerate	4	1	3	1	3	1	3	1	5	1	4	1	4	1	4	2	0.097
2-Oxoglutarate	40	6	28	7	26	6	16	3	45	10	35	9	32	10	20	5	0.044 (0.19)
2-Oxoisocaproate	6	2	6	1	4	0	8	3	7	2	6	2	4	1	9	2	0.48
3-Hydroxybutyrate	380	316	270	184	78	53	414	300	242	217	180	201	160	247	732	1023	0.62
3-Hydroxyisobutyrate	23	5	18	3	13	3	24	11	21	6	18	5	12	3	21	9	0.43
3-Methylhistidine	48	10	35	7	34	10	29	8	48	9	33	9	31	8	27	9	0.56
Acetate	59	32	71	18	74	31	87	39	47	10	60	22	64	32	100	67	0.42
Acetoacetate	153	117	128	72	45	23	248	151	118	99	82	75	88	128	316	356	0.59
Acetone	33	19	24	12	14	3	44	28	33	37	23	15	20	16	67	95	0.92
Alanine	321	74	329	42	348	82	215	24	317	54	383	42	335	67	214	80	0.87
Arginine	73	31	71	16	68	23	65	16	72	29	76	16	73	14	68	22	0.57
Asparagine	43	14	48	13	59	14	44	15	42	12	50	12	48	17	42	15	0.47
Betaine	66	27	49	18	51	17	61	33	92	60	65	28	58	21	52	17	0.53
Butyrate	5	1	5	1	4	1	7	2	6	1	5	1	4	2	7	2	0.72
Carnitine	32	10	32	11	26	4	23	13	32	19	29	8	20	5	23	11	0.41
Creatine	40	13	59	32	44	23	56	34	33	14	71	37	44	14	60	29	0.88
Creatinine	33	5	32	4	33	6	33	2	35	7	33	6	32	4	33	6	0.93
Ethanol	90	38	54	46	76	89	85	101	115	118	93	134	71	68	144	150	0.62
Formate	227	125	224	106	174	105	175	94	194	38	165	71	192	93	157	73	0.54
Fumarate	9	2	7	2	4	1	2	1	9	2	9	4	5	4	3	1	0.31
Glucose	5988	516	7610	1792	5703	809	4357	374	5376	1087	8183	1803	5198	1075	4175	996	0.41

Glutamate	50	15	57	17	53	16	49	22	61	17	65	20	50	21	40	18	0.98 (0.99)
Glutamine	493	58	433	55	467	78	471	64	488	43	469	49	446	48	452	76	0.96
Glycerol	951	143	778	107	833	115	627	186	1074	229	816	251	794	253	634	159	0.87
Glycine	355	42	355	56	374	65	405	61	335	27	365	33	350	54	384	79	0.52
Histidine	86	15	78	16	94	22	66	20	79	16	76	15	75	11	56	12	0.064 (0.19)
Isoleucine	50	21	55	12	45	9	78	36	56	13	59	8	51	18	76	28	0.37
Lactate	6416	1227	7441	1668	7285	1963	1383	423	7080	2764	9547	4819	6536	2291	2203	1963	0.60
Leucine	57	27	68	16	56	15	96	41	63	16	70	8	57	22	91	33	0.61
Lysine	103	34	104	21	103	25	115	33	101	26	114	25	101	25	124	47	0.81 (0.99)
Methionine	39	17	21	4	19	4	21	9	40	18	24	8	23	8	21	6	0.66
myo-Inositol	104	11	85	11	68	12	42	8	102	15	85	18	62	21	47	10	0.86
Phenylalanine	46	13	42	7	46	7	42	13	47	4	44	4	52	15	41	6	0.35
Proline	251	49	215	50	210	66	201	48	248	24	239	28	202	51	185	61	0.99 (0.99)
Pyruvate	278	48	282	49	258	25	71	23	299	101	297	81	276	98	127	121	0.56
Serine	111	19	124	18	126	19	132	19	113	21	131	27	124	28	118	30	0.77
Succinate	23	6	24	10	17	5	7	2	22	8	34	27	13	5	8	2	0.82 (0.99)
Taurine	96	34	86	16	69	11	51	27	98	47	114	48	81	18	59	28	0.42
Threonine	106	41	79	23	78	17	77	14	87	15	78	18	72	15	73	15	0.41
trans-4-Hydroxy-L-proline	74	14	45	8	48	17	37	7	62	17	45	6	42	6	30	7	0.073
Tyrosine	98	31	70	12	56	14	57	11	94	23	84	20	65	13	59	11	0.37
Urea	1640	354	1870	449	1865	558	2871	955	1607	549	1725	459	1645	410	2290	520	0.41
Uridine	4	1	5	1	5	3	7	1	4	3	6	2	5	3	7	2	0.85
Valine	119	42	136	29	125	28	187	71	130	26	145	20	128	26	186	57	0.50

Supplementary Table S9. Mean concentrations of metabolites in infant urine (μM). Since none of the raw p-values was below 0.1, FDR correction and effect size were not calculated. Concentrations are expressed as mean and standard deviation (SD).

	**LC**		**OC**		**p-value**
	**Mean**	**SD**	**Mean**	**SD**	
2-Hydroxybutyrate	5	3	24	34	0.10
2-Hydroxyisobutyrate	14	8	26	19	0.32
2-Hydroxyisovalerate	2	1	3	2	0.25
2-Oxoglutarate	27	15	67	101	0.49
2-Oxoisocaproate	13	7	12	8	0.94
3-Hydroxy-3-methylglutarate	14	9	18	11	0.61
3-Hydroxybutyrate	74	125	2045	5249	0.49
3-Hydroxyisobutyrate	26	18	69	96	0.41
3-Hydroxyisovalerate	18	21	27	30	0.68
3-Indoxylsulfate	69	26	95	48	0.32
4-Hydroxyphenylacetate	56	57	72	77	0.64
4-Hydroxyphenyllactate	11	6	18	17	0.31
Acetamide	13	5	22	21	0.42
Acetate	53	60	130	182	0.82
Acetoacetate	2043	3657	1238	2261	0.82
Acetone	43	44	82	150	0.91
Alanine	11	4	22	27	0.53
Allantoin	809	241	1437	1145	0.29
Arginine	31	11	53	47	0.31
Asparagine	14	6	23	25	0.78
Betaine	17	11	57	82	0.36
Butyrate	18	20	24	23	0.76
Carnitine	24	32	11	17	0.63
Citrate	20	20	30	25	0.59
Creatine	307	525	424	549	0.52
Creatinine	1569	565	3199	3771	0.27
Dimethyl-sulfone	43	31	42	34	0.84
Dimethylamine	95	49	167	178	0.46
Formate	125	100	241	333	0.95
Fucose	12	3	26	21	0.11
Fumarate	3	4	6	8	0.66
Galactonate	227	76	497	573	0.35
Galactose	137	210	200	281	0.56
Glucose	45	29	70	74	0.53
Glucuronate	212	41	304	274	0.75
Glutamate	59	36	80	52	0.47
Glutamine	42	27	124	171	0.18
Glycerol	421	29	461	53	0.12
Glycine	27	8	73	101	0.31
Glycolate	167	81	443	523	0.16

Guanidoacetate	21	9	38	41	0.50
Hippurate	570	365	1111	1222	0.48
Homovanillate	12	5	26	29	0.20
Hypoxanthine	120	52	201	175	0.56
Inosine	11	3	13	6	0.47
Isoleucine	4	2	7	8	0.85
Isopropanol	2	2	6	7	0.36
Lactate	108	148	218	465	0.96
Leucine	3	2	5	6	0.73
Lysine	35	32	37	36	0.74
myo-Inositol	29	14	39	18	0.28
N,N-Dimethylglycine	7	5	27	41	0.71
N-Carbamoyl-beta-alanine	54	40	74	64	0.69
N-Methyl-2-pyridone-5-carboxamide	19	14	24	17	0.79
O-Acetylcarnitine	58	83	80	110	0.25
Pantothenate	26	10	46	35	0.90
Phenylacetate	35	27	32	22	0.58
Phenylacetyl L-Glutamine	1261	659	1214	1178	0.25
Proline	33	11	72	75	0.43
Propylene-glycol	5	3	14	18	0.49
Pyruvate	141	285	18	15	0.39
Quinolinate	34	15	49	29	0.18
Succinate	4	2	22	38	0.69
Taurine	789	285	1054	780	0.32
Threonine	23	15	47	60	0.96
Tiglylglycine	20	8	27	22	0.34
Trimethylamine	2	2	3	2	0.83
Trimethylamine-N-oxide	92	48	116	106	0.70
Tyrosine	15	7	20	14	0.98
Urea	62351	34140	68609	45393	0.38
Valine	5	4	8	7	0.35
